# A New Method for Extracting Skin Microbes Allows Metagenomic Analysis of Whole-Deep Skin

**DOI:** 10.1371/journal.pone.0074914

**Published:** 2013-09-20

**Authors:** Marc Garcia-Garcerà, Koldo Garcia-Etxebarria, Mireia Coscollà, Amparo Latorre, Francesc Calafell

**Affiliations:** 1 Institut de Biologia Evolutiva (CSIC [Consejo Superior de Investigaciones Científicas] - Universitat Pompeu Fabra), Barcelona, Catalonia, Spain; 2 Joint Research Unit on Genomics and Health, Centre for Public Health Research (CSISP) and Cavanilles Institute for Biodiversity and Evolutionary Biology (University of Valencia), Valencia, Spain; 3 Swiss Tropical and Public Health Institute, Basel, Switzerland; Columbia University, United States of America

## Abstract

In the last decade, an extensive effort has been made to characterize the human microbiota, due to its clinical and economic interests. However, a metagenomic approach to the skin microbiota is hampered by the high proportion of host DNA that is recovered. In contrast with the burgeoning field of gut metagenomics, skin metagenomics has been hindered by the absence of an efficient method to avoid sequencing the host DNA. We present here a method for recovering microbial DNA from skin samples, based on a combination of molecular techniques. We have applied this method to mouse skin, and have validated it by standard, quantitative PCR and amplicon sequencing of 16S rRNA. The taxonomic diversity recovered was not altered by this new method, as proved by comparing the phylogenetic structure revealed by 16S rRNA sequencing in untreated vs. treated samples. As proof of concept, we also present the first two mouse skin metagenomes, which allowed discovering new taxa (not only prokaryotes but also viruses and eukaryots) not reachable by 16S rRNA sequencing, as well as to characterize the skin microbiome functional landscape. Our method paves the way for the development of skin metagenomics, which will allow a much deeper knowledge of the skin microbiome and its relationship with the host, both in a healthy state and in relation to disease.

## Introduction

Despite the great interest of skin as an ecosystem, the study of skin microbiome has been recurrently limited by the low host-commensal cell ratio and the high taxonomical divergence among skin sites [1]. The skin is the most external organ in the mammalian body. Its main role is to protect the internal tissues and interact with the external environment, collecting information, preventing loss of temperature and moisture, and defending the body against pathogenis [2,3]. After a long co-existence between host skin cells and the microorganisms that attempt to colonize the body, a set of bacteria consolidated into a skin commensal/mutualistic microbiota. This microbiota is distributed in multiple niches, depending on the amount of nutrients and the physical properties that might result in the most suitable growth conditions for them [4]. The effect of commensal-host interaction may lead to complex behaviors of the whole host system, which, beyond the skin, involves also the immune system [5]. Understanding how the whole system works may lead to crucial knowledge of skin physiology that may be highly relevant to public health and cosmetic pharmacology. Moreover, and under certain conditions (including the host genetic predisposition), the skin microbiota may intervene in the disruption of the skin homeostasis, leading to complex skin diseases, such as atopic dermatitis, psoriasis or eczema. Then, the knowledge of these bacterial disease triggers is crucial to clinical dermatology [6].

Historically, the study of skin microbiota has been severely limited. Culture-based characterization has been shown to be restricted only to the species that grow rapidly under standard laboratory conditions, which are estimated at just ~1% of the species in the skin [7]. Although more complex culture media have been generated, the main solution has come from the culture-independent methods for studying the composition of the microbiota. The most successful approach relies in amplifying the phylogenetic informative 16S ribosomal RNA gene regions from the bacterial community (16S rRNA). This has been applied to most human body habitats, including the gut, oral cavity, or skin, among others [8-10]. Specifically in skin, these pioneering studies have shown a high diversity in microbiota composition, which is highly dependent on the skin physical conditions [11], thus defining a large amount of possible niches. Variability in skin microbiota has been shown to be site-to-site more diverse than between the left- and right-hand sites of the same individual, or between the same sites in different individuals [11].

But second, and more interestingly, the most diverse regions have been shown to be at least as diverse as the gut microbiota. 16S rRNA amplification can identify the species present and their dynamics, but it provides limited or null information about bacterial gene composition, cell function and dynamics, or on microbe-microbe or microbe-host interactions. This limitation has been solved with shotgun high throughput sequencing, pioneered in soil and ocean metagenomic studies [12-14], and applied to functional analyses of the gut bacterial communities [15]. But, despite the success of the functional analysis of other human microbiomes [16-20], this method cannot be applied to the skin microbiome, which occupies all upper layers on the skin, making deep sequencing inefficient and expensive due to the high amount of host DNA being sequenced. Recently a large dataset of human skin microbiome samples has been published under the Human Microbiome Project Consortium [21]. However, those samples have been collected using non invasive superficial methodologies that do not access the full microbiota, or specific subniches such as the eccrine sweat glands or the hair follicle, where a specific diversity may be found and where microbes may be more active [1,9]. Thus, although a wider perspective has been achieved with this methodology, a prokaryotic DNA enrichment is a crucial step for deep skin metagenomic analysis. For that reason, we propose a new approach that allows prokaryotic DNA isolation making subsequent shotgun high throughput sequencing feasible and efficient. This protocol is based on the combination of enzymatic digestion of all bounds between skin cells, and the subsequent separation of cells by size without disrupting their integrity, avoiding the contamination by host DNA. As proof of concept we present the metagenomic analysis of two murine deep-skin microbiomes. Given the long-time use of mouse as a model, and the evolutionary relationship between mice and other mammals, we think that murine validation of this method may extend to human skin samples, and may also allow retrieving higher quality, more relevant information than that obtained with a less invasive sampling process. 

## Materials and Methods

### Mouse skin sampling

Eight healthy C57BL/6J mice of 8 weeks of age were included in this study. All steps of animal handling were carried out to minimize the stress of the animals while keeping them isolated to reduce possible microbiota share. All mice were euthanized through cervical dislocation, according to a local IRB-board (PRBB, IACU committee) approved protocol, and a region of 3x3 cm was excised from the dorsum-lumbar region, using a sterile blade, and frozen in liquid nitrogen to preserve the integrity of the skin. The samples were subsequently split using a 6 mm punch blade and stored at -80°C until further experiments. All mice followed the same feeding rate, and were born and housed under the same conditions in an animal core facility, accredited by AAALAC International. Of these individuals, six were used to test bacterial diversity (named B1 to B6 when used for bacterial enrichment and T1 to T6 for total DNA extraction) and two, namely Sample A and Sample B, for metagenomic analysis.

A negative control was included in the analysis to test all possible materials and reagent contaminations.

### Procedure

**Figure 1:**
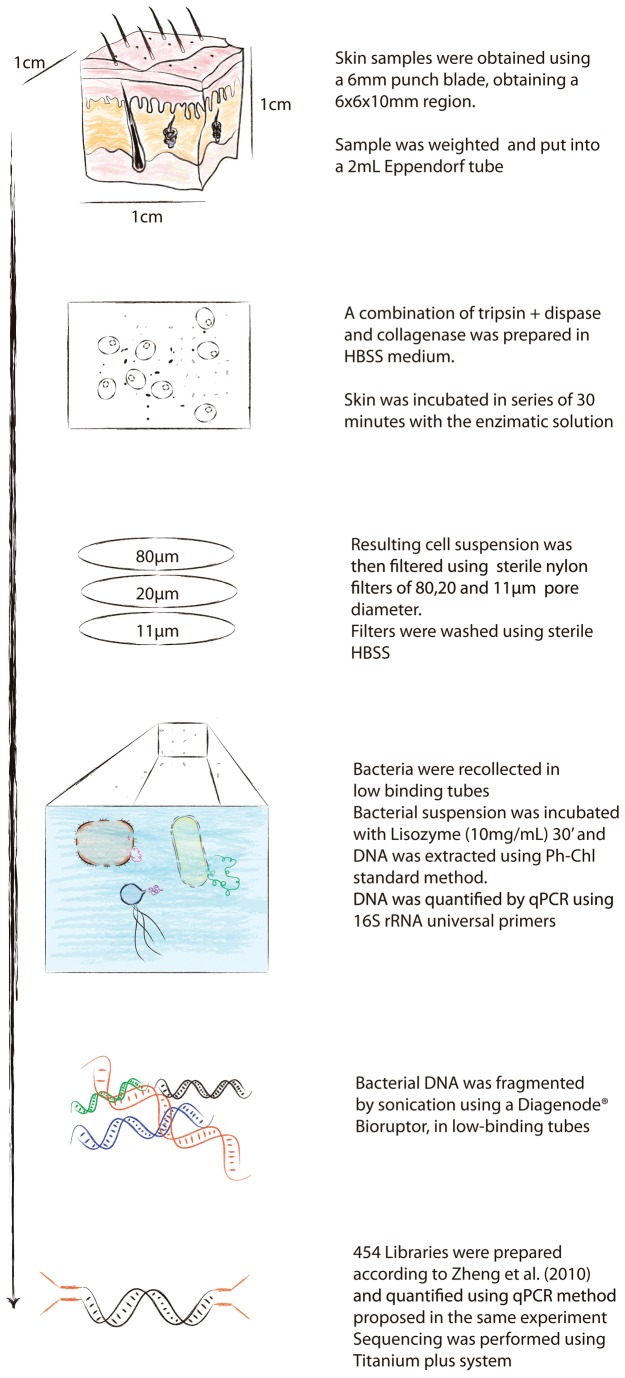
Schematic description of the proposed method.

The proposed method and its validation is shown in File S1. A graphical diagram of the procedure can be seen in Figure 1. In brief, a 6mm skin sample was digested three times using a buffered enzymatic solution in constant shaking. The resulting solution was sequentially filtered using sterile nylon filters, eliminating all the host cells.

To avoid mitochondrial contamination (see File S1 for further information), mitochondria were eliminated through flow cytometry, leaving only cells with a genome size larger than 0.5Mb.The remaining cells were then digested and DNA was collected using a standard Phenol-Chloroform method.

The method was validated in two different aspects: host, contaminant, and bacterial DNA was quantified, and the taxonomic composition obtained was compared with that obtained with a standard DNA extraction without microbial DNA enrichment.

Finally, the method was tested by performing a metagenomic analysis of two mouse skin samples, presenting for the first time a functional assessment of the deep skin microbiota.

## Results

### Microbial and host DNA detection by standard PCR in the proposed method

Skin samples were collected from genetically and environmental homogeneous mice to reduce as much as possible the environmental variability that could affect the diversity of the skin microbiota. Our method resulted in the successful isolation of around 5 ng of DNA (ranging from 3.2 ng to 8.7 ng). We tried to amplify a host gene, IRGA6, and the bacterial 16S rRNA to assess the ability of our protocol to substantially reduce the amount of host DNA. No band was observed in the IRGA6 amplification, but all of 16S rRNA amplification resulted in one size-specific band (Figure S1). Control DNA extraction, as expected, resulted in a specific band in both 16S and IRGA6 genes. No visible bands were observed in any of the samples or extraction methods for the primate-specific NPIP gene used to monitor human contamination. The mock sample used to check possible reagent contamination failed to amplify for any of the three methods. Specific negative controls for each primer pair also failed to produce any visible amplfication.

### Host DNA reduction test by qPCR

A qPCR experiment was performed using the same genes and the same samples, to measure the actual amount of host DNA in each method (Figure S2). Cq values are shown in the Table S1. The quantitative PCR analyses showed the same trend than the previous standard amplification protocol. Although in most cases bacterial DNA yield was reduced in the method proposed due to the retention of bacteria by nylon filters, no statistical difference was observed (Chi-square p-value=0.089), while host DNA yield was significantly reduced beyond the limit of detection of the termocycler (Cq > 35) in all cases (p-value<10^-5^). If we consider that the standard curve minimum was set at 10^-4^ ng for host DNA, with a Cq of 32, values of Cq over 35 suggest a complete elimination of host DNA from the sample. The mock sample produced Cq values that were similar to those of the negative control (16S amplification Cq = 33.08/33.46, IRGA6 amplification Cq = 37.27/36.89). The differences between the mock sample and the negative control would come from the different reagents in both samples, since the mock sample followed the same process than the rest of the samples while the negative control was performed using nuclease-free water as template. We observed that 16S amplification always resulted in a negative amplification over the 30th cycle, which implies that the 16S amplification of mock sample may be considered as negative [22].

### Diversity bias assessment

Taking into account the bacterial DNA yield reduction, the following question was to assess whether the reduction was associated with a loss of diversity in our skin bacterial community. The bacterial 16S rRNA gene was amplified in all samples and extraction methods and sequenced using parallel-tagged 454 titanium technologies, resulting in 654,472 sequences with lengths ranging between 50 and 850 nucleotides. Of these, 645,474 reads (98.6%) passed all filters of length and quality and were used for further analyses. Pooled reads were split by sample resulting in 19 samples with reads, ranging from 1,000 to 22,000 reads per sample. The mock sample just yielded 17 low-quality reads, and was thus eliminated from further analyses. Given the absolute differences between samples, a random subsampling without replacement was performed, and repeated 200 times; statistical analyses were corrected with the false discovery rate approach.

At the same time, pooled sequences were clustered using the greedy algorithm implemented in CD-HIT resulting in 8,696 and 1,275 OTUs at identity levels 99% and 97%, respectively [23]. A phylogenetic tree was constructed using the prior probabilities and seed tree provided by RaxML using the default configuration [24]. As our alignment contained only 181 phylogenetically informative positions, which are not sufficient to classify the 1,275 phylotypes, we used the reference sequences from the NCBI to construct the tree. Taking into account the stringency of our assignment, the resulting tree can be considered representative and more realistic than the tree resulting from using only the 181 informative positions. Figure S3 shows the phylogenetic architecture of our community, where *Proteobacteria* were abundant, (mean frequency,79%; standard deviation,8%; Figure S4). An average 53.7% of the *Proteobacteria* reads belonged to *gammaproteobacteria* (compared to an average 38.4% of *betaproteobacteria*, 7.3% of *alphaproteobacteria*, and 0.6% of all other classes). But in terms of taxonomic diversity, we observed that most samples tended to carry more species belonging to the *betaproteobacteria* genera than to the rest of *Proteobacteriaclasses* . The rest of OTUs were spread in several bacterial phyla, but most sequences were assigned to *firmicutes, actinobacteria*, and *bacterioidetes*, the other three principal phyla previously associated with mammalian skin microbiota [11].

**Figure 2:**
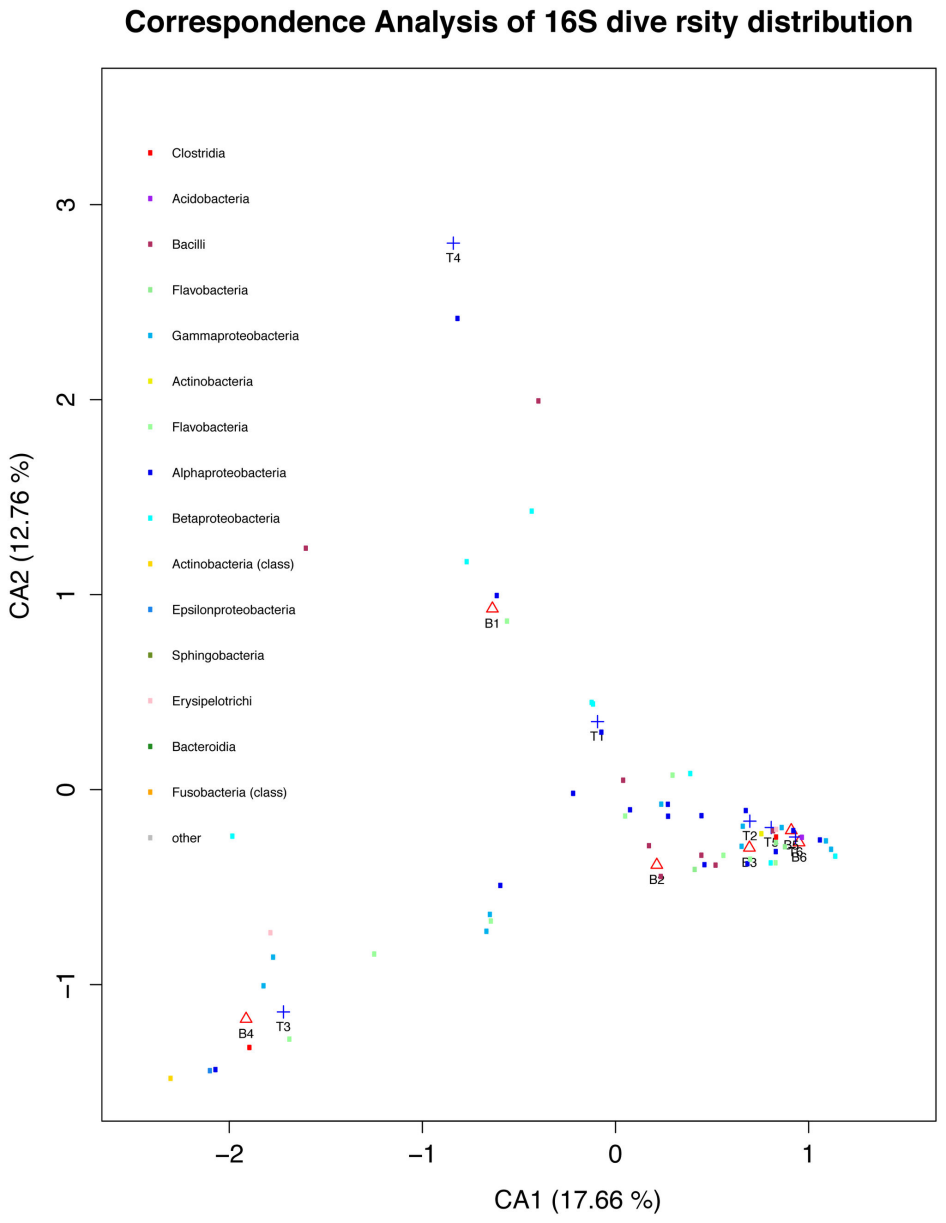
Canonical Correspondence Analysis (CCA) of the bacterial diversity in skin in Standard and Proposed methods. Methods are labelled B for Bacterial Enrichment extraction and T for Total DNA extraction.

To further test whether the phylogenetic composition retrieved may be altered with this method, the taxonomic abundance was compared between the new and the standard protocols. Interestingly, the bacterial enrichment method yields a higher number of phylotypes compared with the standard method. The same trend was also observed with Shannon Diversity and Rarefaction curves(Table S2 and Figure S5). However, differences among groups are not statistically significant (Wilcoxon test. p-value = 0.327). Samples appear to be different, but they do not cluster by method. Canonical correspondence analysis (CCA) shows no separation by sample or extraction method (Figure 2) [25-27]. Principal Coordinate Analysis (PCoA) analysis shows concordant results (Figure S11). We did not observe any statistical significant difference either among samples (ANOVA, p= 0.13) or among methods (p = 0.29), based on distance dissimilarities. Moreover, non-metric multidimesional scaling (NMDS) showed a complete scattering and admixture of the samples showing also no aggregation by method (Figures S6 and S7). Taxon abundances were not statistically significantly different among samples (p =0.375) or among extraction methods (p =0.795).

### Metagenomic library preparation and proof of concept

Two independent samples were extracted according to the method proposed. Sheared DNA was blunt-end repaired and ligated to the adaptors. Library quantification was performed with qPCR, and the correct amount was used for emPCR and sequencing according to the method adaptation from Zheng et al [28], resulting in 60,488 (sample A, MG-RAST accession number 4496968.3) and 65,647 reads (sample B, 4496969.3).

**Figure 3:**
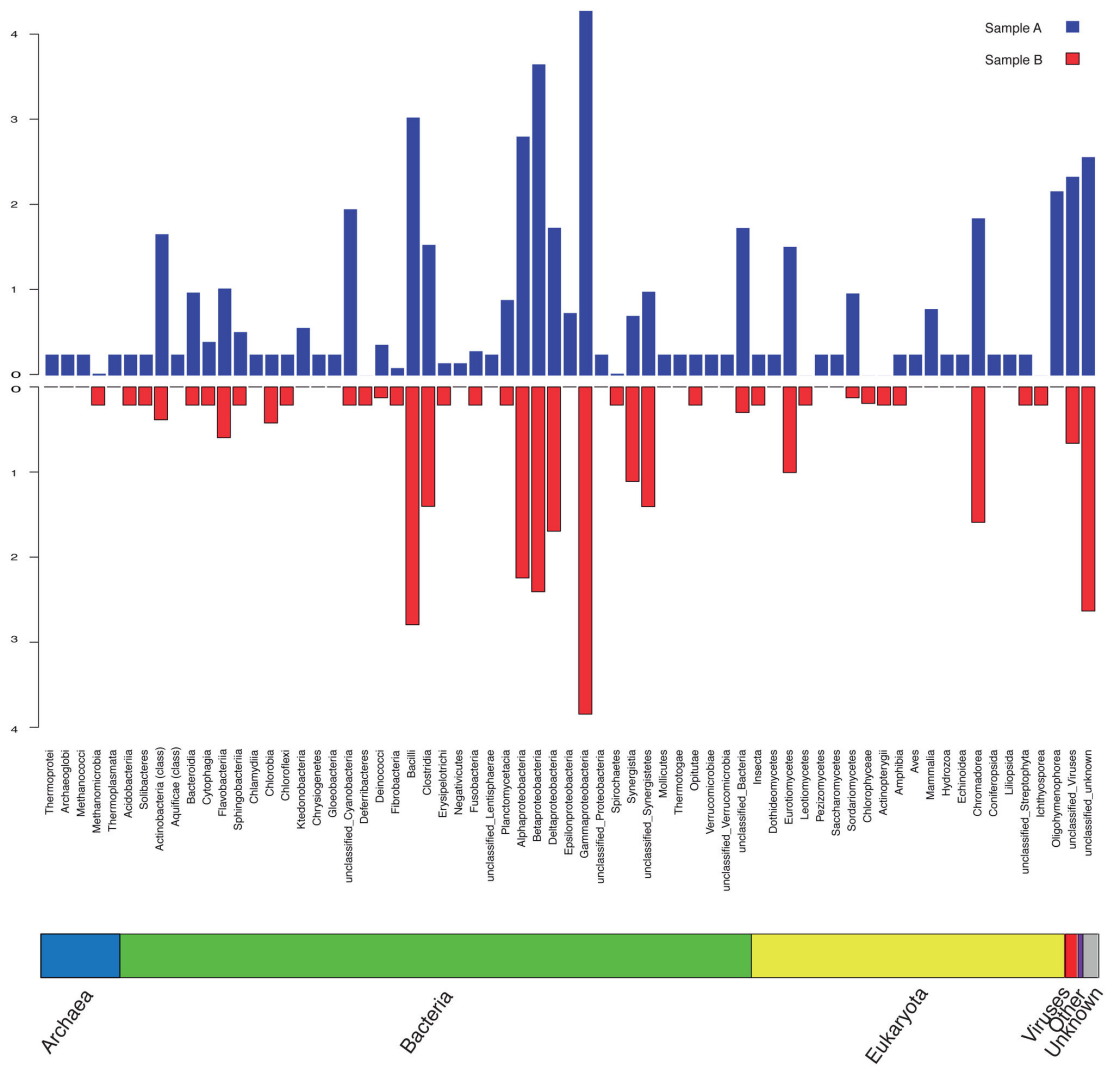
Comparison of the relative abundance of bacteria in skin metagenomic samples. 454 reads were split by taxonomy at class level. Relative abundance was log transformed to reduce the drastic differences among taxa assignation. Samples A (red) and B (blue) are faced by taxa, to facilitate the comparison.

In both samples, 95% of reads were assigned to bacteria (Figure 3), 1.92% to other commensals (fungi, arthropods), and 0.02% to host DNA. Thus, the enrichment protocol has resulted both in a total amount of DNA (~ 5 ng) and in a proportion of microbial DNA that make a metagenomic analysis feasible.

Most of the bacterial reads belonged to the Proteobacteria phylum (85% of reads in sample A and 88% in sample B) whereas the presence of the rest of phyla was quite limited, except for Firmicutes. These proportions are similar to those observed with 16S rRNA sequencing (Figure S4). In lower taxonomical levels, a difference appears between samples: Sample A seemed to be less diverse than sample B. Although in both samples Gammaproteobacteria was the dominant class, the presence of other classes was quite limited in sample A, whereas in sample B the presence of other classes, such as Betaproteobacteria, was higher. The same pattern occurred at lower taxonomical levels. For example, 74% of the reads of sample A were assigned to the order Pseudomonadales and only 48% in sample B. In this sample the presence of Burkholderiales and Enterobacteriales was higher (16% and 15% respectively) than in sample A (around 2% both). At family and genus levels, Moraxellaceae (72% in sample A and 48% in sample B) and *Acinetobacter* (71% and 45%), respectively, were the dominant taxa. These results agree with the 16S taxonomical distribution for both methodologies. To confirm the methodology validation, samples A and B were processed with both methodologies and amplified for 16S rRNA. Taxonomic distribution was compared with the 6 previous samples used to validate the method. 16S rRNA amplification of samples A and B produced a taxonomic disribution that was similar to the other samples, supporting the previous validation (Figure S12). We obtained the taxonomic distribution in samples A and B with two methods: metagenomics and and 16S amplification. 16S rRNA amplification of samples A and B retrieved 32 and 41 different genera, compared to 40 and 76 different bacterial genera obtained by metagenomic sequencing after applying the same subsampling approach previously mentioned. In contrast, raw metagenomic data was assigned to 177 and 368 bacterial genera, highly increasing the diversity observed by 16S rRNA amplification. Shannon diversity in 16S was statistically lower than that observed by metagenomic characterization (1.14 vs 2.46 after random bootstrap subsampling with 200 replicates; two tailed t-test. p = 1.45×10^-8^). Taxonomic assignation of metagenomic samples A and B in NMDS shows a clear separation from those processed with amplification methods (Figure S13). A large number of taxa was present exclusively in metagenomic datasets, compared to the 16S rRNA datasets. 16S amplification of samples A and B clusters together with the rest of the samples amplified, while metagenomic datasets from sample A and B behave independently (Figure S14).

In addition, the metagenomic approach allowed discovering non-bacterial species. 1.8% of all the dataset was assigned to Eukarya, and of those, 56% was assigned to Fungi, 13% to Arthropoda, and less than 20% to Chordata. Fungi classes such as Dothideomycetes [29], Eurotiomycetes [30], and Leotiomycetes, among others (Figure 3), have been observed in our dataset, and have previously been isolated from mammalian skin. Interestingly, a wide range of plant classes were also retrieved.

Functional analysis was performed using hidden Markov models (HMM) constructed with the multiple alignments of the functional categories from the COG [31] and eggNOG [32] orthology databases. At COG 1 level, unknown function or general function prediction, both categories assigned to unknown function categories, were the most frequent function in both samples (53.3% and 27.2% each). Within the known functions, catabolism, as expected, was the most frequent, comprising mostly protein and carbohydrate degradation (11.93% and 29.85% respectively). Other categories such as nitrogen metabolism or respiration were also enriched, although to a lesser degree. Although in general, Sample B was more diverse than Sample A, similar trends were observed in both samples.

**Figure 4:**
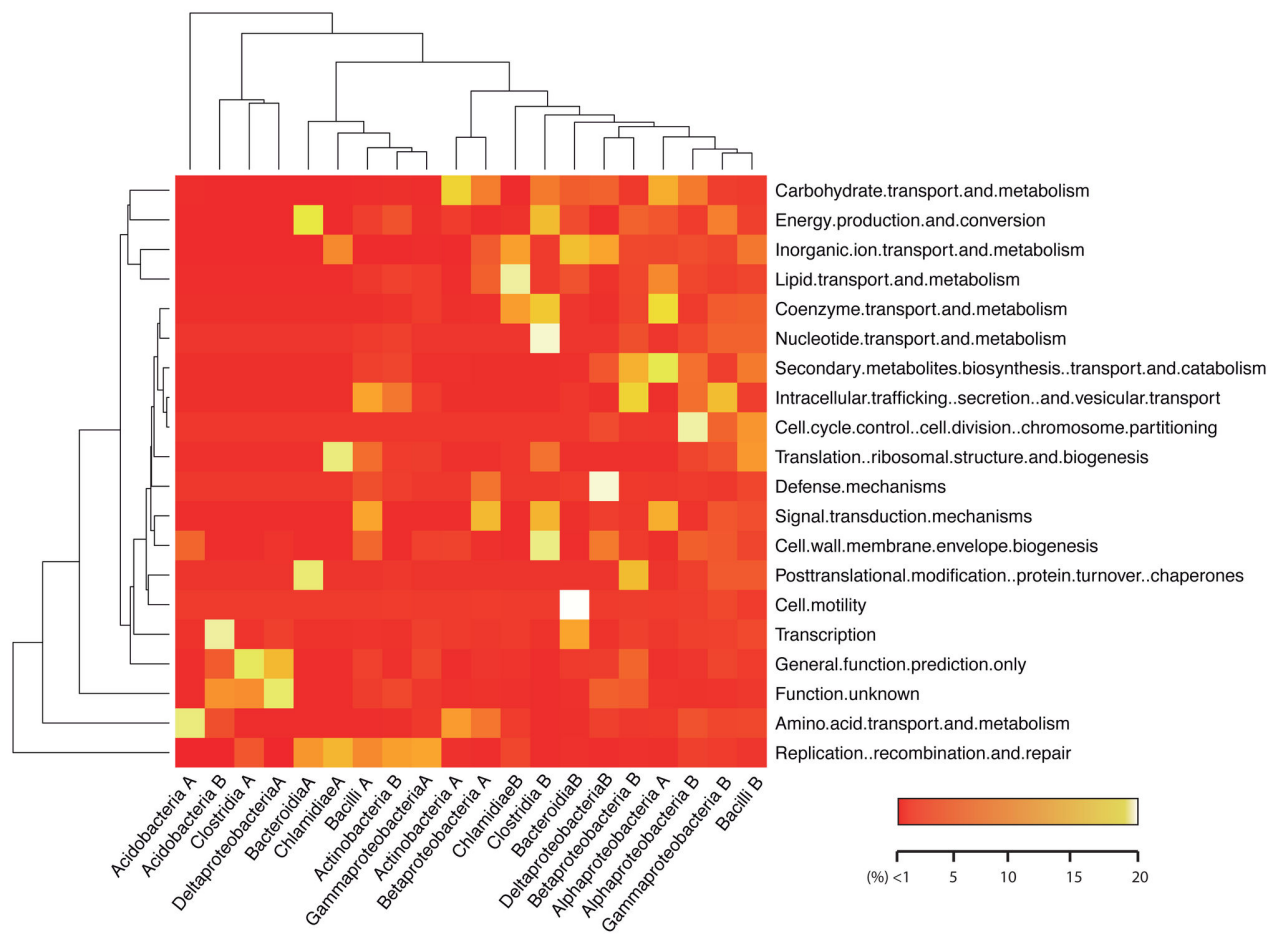
Metagenomic profiles of Skin metagenomes, split by taxa. Classification was made first by taxonomic assignation using PhymmBL, and then functional classification was based on EggNOG using HMM classification. Counts were normalized by sample and taxa using log transformation. The color gradient indicates the level of representation for that taxa. Hierarchical clustering was performed by function and taxon.

Following on the functional analysis, datasets were split taxonomically, to assess similarities and differences at functional level for each taxonomic subcluster. The functional distribution in both samples was different due to the different rate of reads assigned to the unknown function cluster, but at taxonomic subsystems the differences were more dramatic. Relative abundance patterns were different among taxonomic subsamples (Figure 4). Although we observe that the basic functional trends are present in all class clusters (including replication, protein and energy production), the most abundant function present in each sample and each taxonomic cluster was different. For instance, genes assigned to motility were enriched in the Bacteroidia cluster from sample B, but not in other class clusters of the same sample or in any of the taxonomic clusters from sample A. In general, the functional signal from sample B was broader and stronger than that from sample A, in agreement with the reduced diversity observed. Sample A was enriched in replication, recombination and repair genes, and this trend was shared by distant taxonomic groups, suggesting a possible environmental effect affecting the functionality and diversity of skin microbiota of mouse A. But differences were observed even in taxonomic groups with a wide range of functions observed, such as Alphaproteobacteria. Taxonomic clusters were analyzed using PCoA (Figure S8) and NMDS (Figure S9). In both cases, they showed no association by taxon. While Sample B aggregated around the centroid, suggesting that functions were homogeneous at all taxonomic levels, sample A was more spread, meaning that some functions were associated with particular taxonomic groups.

## Discussion

Here we present a new method, based in cell and molecular biology techniques, to sharply reduce the amount of host DNA from a skin biopsy sample obtained with standard procedures. We have validated the new method in terms of isolation, efficiency and taxonomic bias inferred by qPCR results. With this protocol we have obtained 95% of bacterial DNA recovery compared to less than 1% expected by direct skin biopsy DNA extraction and sequencing (standard procedures). On average, we obtained 5 ng of bacterial DNA, which is sufficient to prepare a sample using the protocol by Zheng et al. [28]. Although pool sequencing was performed to obtain equimolar quantities of each sample, we observed a bias on the resulting read count for each sample. Keeping in mind that the quantification of samples prior to the pooling was done by qPCR, the variance in sequence retrieval difference is likely to be due to stochastic variables of the sequencing procedure or differences in the efficiency of the fusion primers used for the experiment. Nevertheless, the taxonomic structure was maintained, even recovering more diversity than in standard procedures, including eukaryotic members of the microbial consortium associated with the skin. The importance of detecting fungi or arthropods is clear since they are also commensals, and may also be involved in the maintenance of the skin homoeostasis. It is important to note that we could detect those organisms even with the tight particle size restrictions imposed by our method. Our results are in agreement with the phylogenetic diversity observed in other skin microbiome 16S rRNA studies [9,33], which implies that our method does not introduce any major taxonomic bias. Moreover, our study unveils that mouse skin carries a smaller number of phylotypes, dominated by *Proteobacteria*, in comparison with other environments such as gut, where *Firmicutes* and *Bacteroidetes* are the most abundant, or human skin where the predominant phylum seems to be variable according to the skin niche analyzed [11,34,35]. Rarefaction curves (Figure S10) show lower numbesr of phylotypes per 1000 reads, with a gentler slope those observed in gut [36]. These results agree with previous 16S-based works [9,10,33], implying that the use of this method does not entail a bias and, in addition, new taxa may be discovered.

This protocol allows not only the taxonomical characterization of the skin microbiota but also to explore the functionality of that microbiota and the possible biochemical and molecular relationships among its individuals and between the microbiota and the host. Two samples were processed using our method, and were fully sequenced to see differences in taxonomy and function, compared to the results obtained from 16S rRNA analysis; for the first time, a functional analysis of deep-skin microbiota can be produced. Besides the differences in the taxonomic distribution, one can also uncover previously unknown functional trends that occur on the skin ecosystem, which may have an important impact in health and cosmetic pharmaceutics.

The different functional databases used produce different results based on specific biases of their original datasets, and therefore we used different databases to characterize the functional annotation of the skin metagenomes. Although catabolism was the most abundant functional category in our datasets, other traits can be considered more interesting; categories such as respiration, nitrogen metabolism and other clusters associated with aerobiosis were enriched. Amino acid metabolism and protein degradation were also important in our dataset. However, this may be related to taxonomic abundances and, then, it must be taken carefully. Nevertheless, all these trends are in agreement with the particularities of skin as an ecosystem, considering the continuous skin replacement and the direct contact with the atmosphere.

One of the most interesting questions that can be addressed using metagenomic data is the functional niche occupied by each taxonomic group, which has been called the “who does what” question. However, despite the fact that this question can only be answered with transcription data, one may approximate the analysis to the “who can do what”, with functional analysis of metagenomic data. Even if our main goal was to obtain a proof of concept for the new methodology proposed, the functional analysis applied to taxonomic specific data showed interesting results. In some of the taxonomic clusters we observe different functions associated to them in either sample. This functional divergence at the same taxonomic level suggests that although the main functional roles are present in those samples, and maintain the specific functional repertoire associated with the skin ecosystem, the functional roles taken by many taxa were different in each sample. These differences may be due to specific events that only occurred to each mouse, specific metabolic or immune characteristics of each mouse or it was due to some bias produced by the procedure in that specific sample. Further sampling and research is needed to understand the skin from an ecosystem point of view.

Using this protocol, the level of knowledge of the skin microbiota may be brought to the level of the widely analyzed gut and oral microbiota, in which microbiota has been related to physiological and pathological changes in the host [37-39]. The gut microbiota has been related to immune system and gut differentiation [38,40-42] and chronic inflammatory diseases [18,43,44]. With this method we open a new door to explore the skin microbiota and its possible implications in complex behaviors of skin, including skin complex diseases, as it has occurred with the gut and oral microbiomes.

However, one of the most striking points of this methodology is the facility to translate it to other host-microbiome scenarios. The most interesting case, and the easiest to apply, is the gut. It is understandable that none of the metagenomic studies performed in gut has been performed in biopsies, given the low bacterial/host DNA ratio in them. This is why, up to date, all gut metagenomic studies have been performed in feces. The same problem occurs in the oral ecosystem, where most studies have been performed in swabs and plaque extraction, where host cell loads are low. In fact, the most recent paper from the Human Microbiome Project Consortium was performed using swabbing methods in 18 regions [21]. But the bacteria in close relationship with the host cells, those in constant contact, are the most important in the progression and the maintenance of the ecosystem homoeostasis, and should be studied in more detail.

Although all the previous ideas are only suggestions, we think that the method can be a strong advance in the field of metagenomics and all its variants. Working with deep epithelial biopsies would shed light into the host-microbiota relationships in any conditions, pushing further the studies of host-microbiota interactions and its relationship in health and complex diseases.

## Data Access

16S rRNA data from the 12 samples is deposited on the MG-RAST database with public accession numbers from 4523521.3 to 4523536.3. Metagenomic data from both samples is deposited on the MG-RAST database with the accession numbers 4496968.3 and 4496969.3.

## Supporting Information

Figure S1 **Standard Amplification of Host, Bacterial and Contaminant DNA.** Gel visualization of the bacterial (16S), host (IRGA6) and human contaminant (NPIP) gene standard amplification in 12 independent samples, plus a host and a human contaminant controls. Each amplification was performed independently with its own negative control for 16S. Host and contaminant controls were tested independently with their own negative control.
(PDF)

Figure S2 **Quantification of host and bacterial DNA by qPCR.** Amplification curves of 16S rRNA (red) and IRGA6 (green) genes were tested and compared among standard and proposed method. Two equivalent samples were processed to render the amplification curves comparable. Differences on Cq can be interpreted as differences in the amount of DNA on that sample for a given DNA type.
(PDF)

Figure S3 **Phylogenetic tree reconstruction by RAxML.** Reference phylogenetic tree obtained by Maximum Likelihood analysis of the alignment of 16S reference sequences obtained from RDP and selected by similarity, using CD-HIT. Triangle height indicates phylogenetic diversity within each group. On the right hand panel, relative abundances of each taxon.
(PDF)

Figure S4 **Comparison of the relative abundance of bacteria in skin samples using both methodologies of extraction.** 16S DNA sequences were assigned to the genus level. Only genera with more than three sequences assigned were used for the analysis. Then whole information was clustered to class level. Methods are named by the capital letter (B from Bacterial Enrichment extraction and T from Total DNA extraction).
(PDF)

Figure S5 **Phylogenetic diversity rarefaction curves for the different samples and methods.** Rarefaction curves based on phylogenetic clusters at 97% similarity. Curves were normalized to 10,000 sequences according to the expected slope.
(PDF)

Figure S6 **Nonmetric Multidimensional Scaling Analysis of 16S rDNA diversity in standard and bacterial Enrichment methods.** First, NMDS was constructed for Sample diversity, using a Manhattan distance matrix and 20 replicates. The resulting rank matrix was reduced to two dimensions, which were used to construct the graph. Colors (blue and red) separate both methods following the same pattern as in figures in the main paper. NMDS matrix was constructed for taxa diversity using the same distance algorithm. Both matrices were normalized one to each other to be comparable. Taxa diversity was plotted by name. Letter size was associated with the relative mean abundance of each taxon. Color was selected by class, using a phylum-based chart.
(PDF)

Figure S7 **NMDS analysis of 16S rDNA diversity in standard and bacterial enrichment methods (alternative view).** Additional perspective of the NMDS taxonomic distribution. Taxonomic categories (genera) are colored according to their class category classification.
(PDF)

Figure S8 **Correspondence Analysis (CoA) of the bacterial function in skin samples, separated by taxa.** Samples are represented by color (Sample A in red, sample B in blue), and functions by letter, using the standard eggNOG function category code.
(PDF)

Figure S9 **Non-metric Multidimensional Scaling analysis of the taxonomic-based functional diversity for metagenomic samples.** Manhattan Distance matrix was constructed for each of the sample-specific taxonomic clusters. Rank classification and dimension scaling was constructed based on the distance matrix. Samples are represented by colour (Sample A in red, Sample B in blue).
(PDF)

Figure S10 **Phylogenetic diversity rarefaction curves for metagenomic samples.** Rarefaction curves were constructed by random sampling of reads with taxonomic assignment, using the taxonomic information previously obtained.
(PDF)

Figure S11 **Principal Coordinate Analysis of 16S taxonomic distribution abundances.** Bray-Curtis Distance matrix was calculated based on the resampled taxonomic abundances at genus level, for each sample from the validation step. Genus were colored according to their ‘class’ category. Extraction methods were labelled with a capital letter (B as bacterial enrichment and T as total extraction).
(PDF)

Figure S12 **Principal Coordinate Analysis of 16S taxonomic distribution abundances.** Samples A and B were processed according to the validation step of 16S rRNA amplification, using both methodologies. The Bray-Curtis Distance matrix was calculated for each sample, and dimension scaling was performed using the PCoA method. Samples were named and highlighted by method (bacterial enrichment method in blue, total DNA extraction in red)..
(PDF)

Figure S13 **NonMetric Multidimensional Scaling taxonomic analysis of 16S and Metagenomic datasets.** The metagenomic method was compared to 16S rRNA amplification in the same samples. In light blue, we see the Samples A and B metagenomic datasets and in dark blue the 16S amplification of the same samples. Color dots show taxonomic entities at genus level, colored by class. As control we have included the 16S rRNA amplifications of the remaining samples included in the work to compare the relative positions on the 2-dimensional space of the metagenomic and the 16S rRNA methodologies.
(PDF)

Figure S14 **16S rRNA and metagenomic data comparison and between-class analysis of Taxonomic assignment.**
A) Principal Coordinate Analysis of Sample dissimilarities. The Bray-Curtis distance was calculated and used to calculate the principal coordinates. Color tags and names were used to differentiate between the three different methodologies: Metagenomic Assignation (in black), bacterial enrichment (blue) and total DNA isolation (red).
B) Principal Coordinate Analysis of taxa dissimilarities. Points show the genera distribution across the dimension-reduced space, and color tags were used to differenciate taxonomic units (at class level).
To construct the clusters in both cases, the Calinski-Harabasz index was used to estimate the optimal number of clusters. Cluster analysis was performed using the *cluster* package in R. Between-class analysis was performed to assess the variance from the centroid point for each cluster. Graphical interpretation was performed using the package *ade4*.
(PDF)

File S1 **Supplementary materials and methods.**
(DOCX)

Table S1 **qPCR values for bacterial 16S rRNA and the murine IRGA6 gene. B, samples enriched for bacteria; T, total extraction samples.**
(DOCX)

Table S2 **Diversity measurements of 16S rRNA sequences.** Diversity indexes (Shannon diversity index, Chao1 Richness and ACE) were calculated for each sample given a family-based abundance table. B, bacterial enrichment samples; T, total extraction samples; Samples A and B for metagenomic samples.
SE, Standard Error; ACE, Abundance-base Coverage Estimator. Undefined (NaN) values appear when all rare taxa are only assigned as singletons.
(DOCX)
