# Supplementary material for: A New Method for Extracting Skin Microbes Allows Metagenomic Analysis of Whole-Deep Skin
Source: PLoS One. 2013 Sep 20;8(9):e74914. doi: 10.1371/journal.pone.0074914 (PMC3779245; doi:10.1371/journal.pone.0074914)
Supplement: Figure S1 — Standard Amplification of Host, Bacterial and Contaminant DNA. Gel visualization of the bacterial (16S), host (IRGA6) and human contaminant (NPIP) gene standard amplification in 12 independent samples, plus a host and a human contaminant controls. Each amplification was performed independently with its own negative control for 16S. Host and contaminant controls were tested independently with their own negative control. (PDF) [file pone.0074914.s001.pdf]

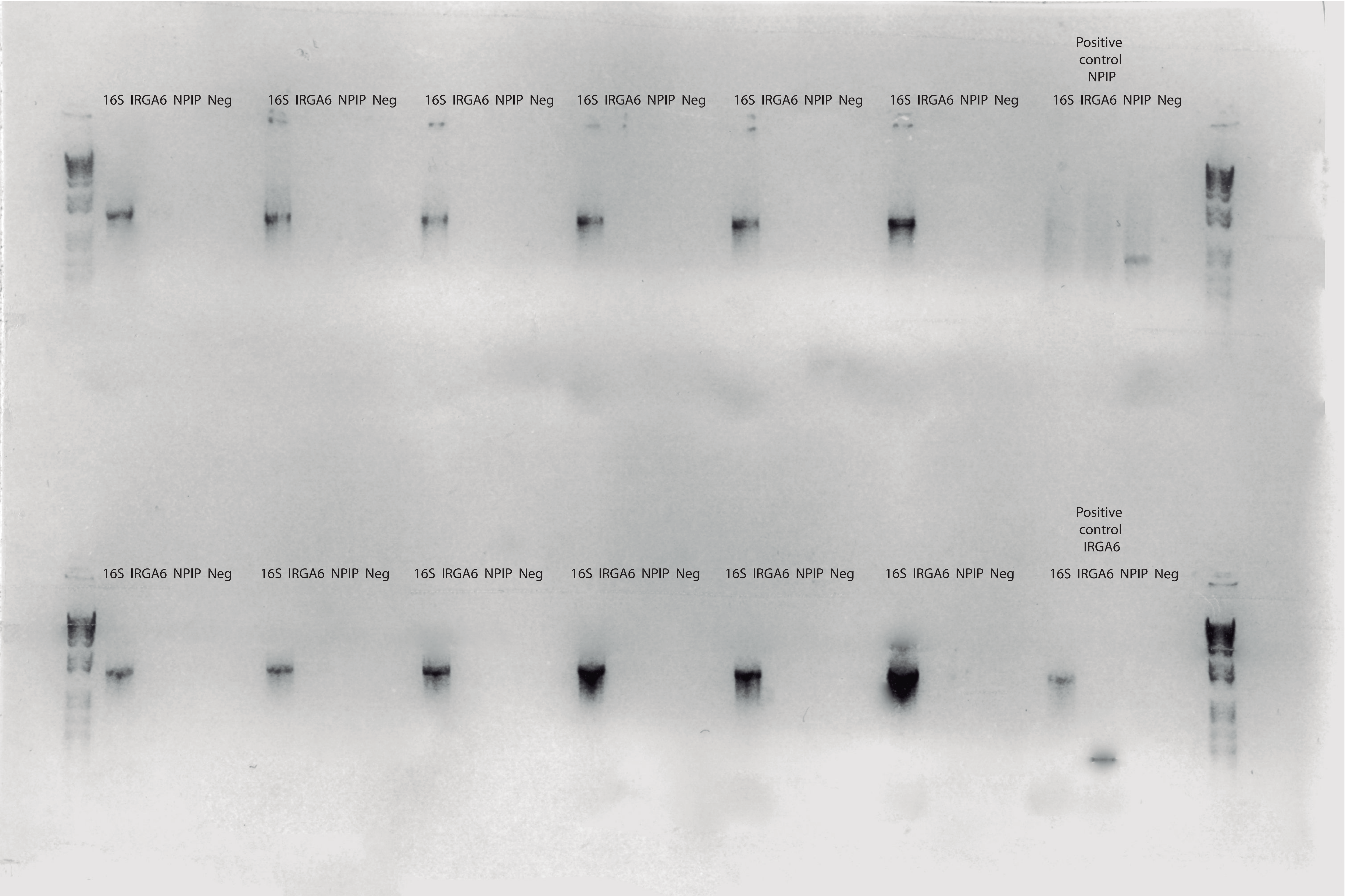

Positive control NPIP
16S IRGA6 NPIP Neg
16S IRGA6 NPIP Neg
16S IRGA6 NPIP Neg
16S IRGA6 NPIP Neg
16S IRGA6 NPIP Neg
16S IRGA6 NPIP Neg
16S IRGA6 NPIP Neg
Positive control IRGA6
16S IRGA6 NPIP Neg
16S IRGA6 NPIP Neg
16S IRGA6 NPIP Neg
16S IRGA6 NPIP Neg
16S IRGA6 NPIP Neg
16S IRGA6 NPIP Neg
16S IRGA6 NPIP Neg
