# Supplementary figures and images for: A New Method for Extracting Skin Microbes Allows Metagenomic Analysis of Whole-Deep Skin
Source: PLoS One. 2013 Sep 20;8(9):e74914. doi: 10.1371/journal.pone.0074914 (PMC3779245; doi:10.1371/journal.pone.0074914)

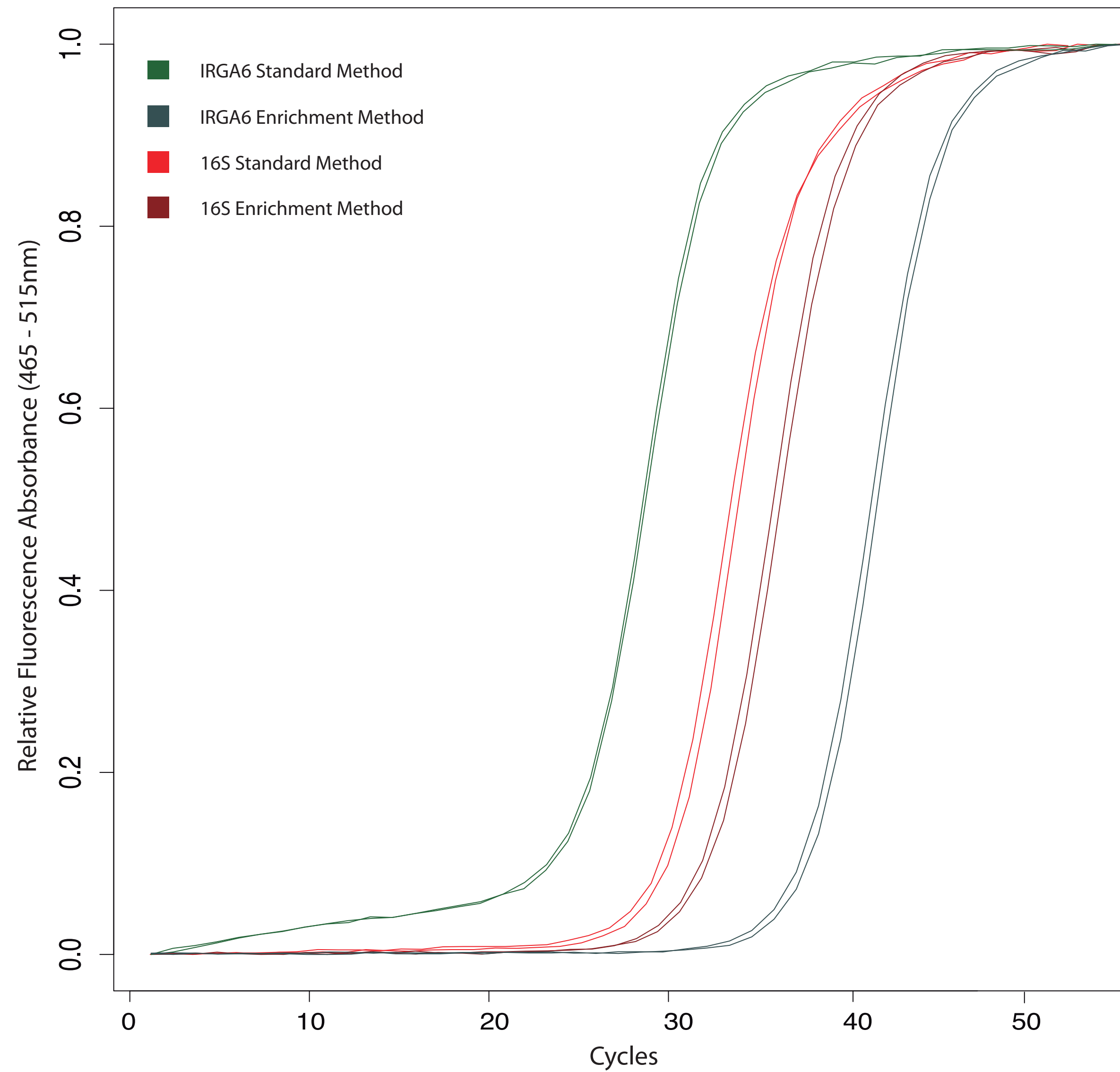

Supplement: Figure S2 — Quantification of host and bacterial DNA by qPCR. Amplification curves of 16S rRNA (red) and IRGA6 (green) genes were tested and compared among standard and proposed method. Two equivalent samples were processed to render the amplification curves comparable. Differences on Cq can be interpreted as differences in the amount of DNA on that sample for a given DNA type. (PDF) [file pone.0074914.s002.pdf]

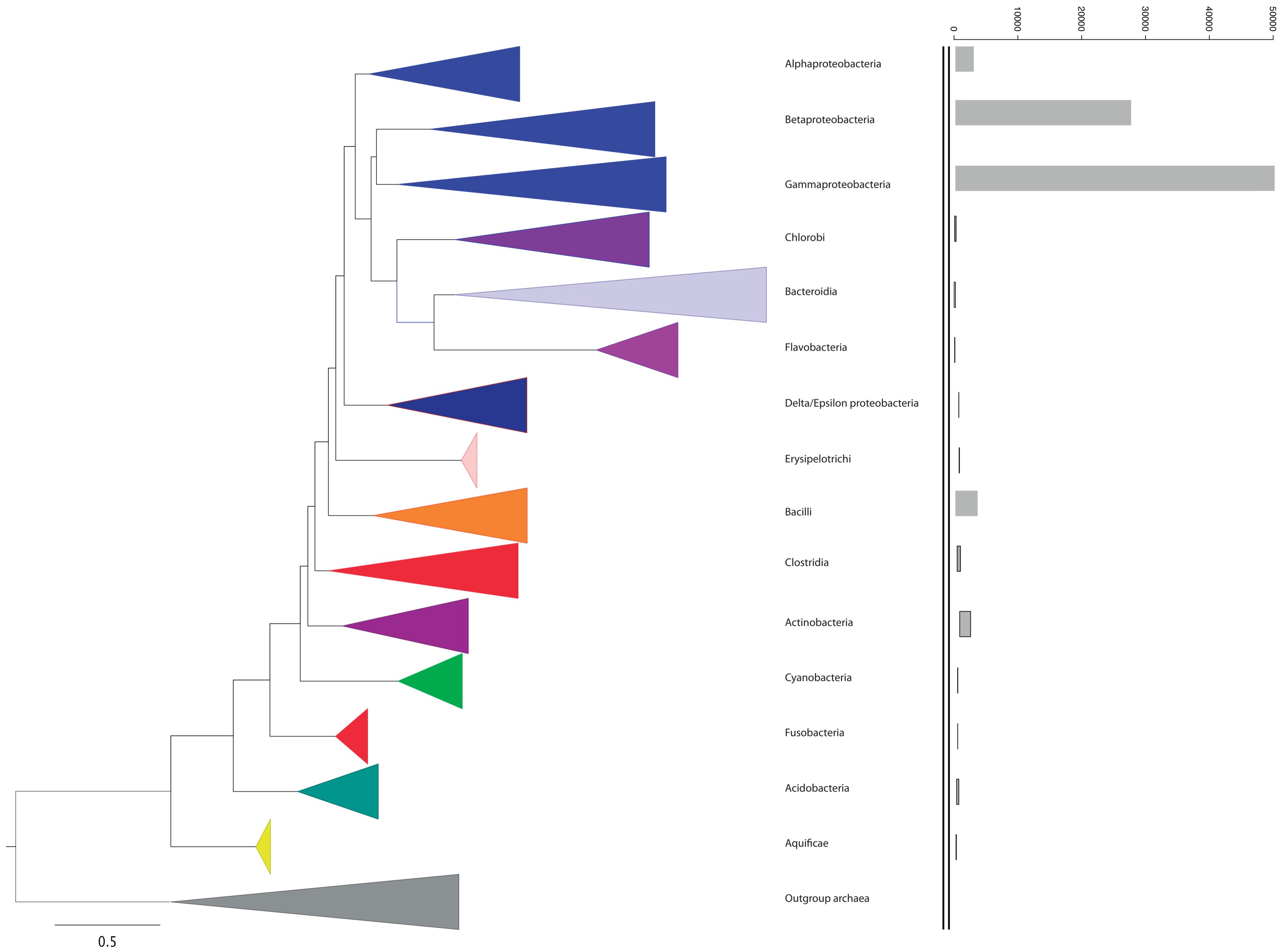

Supplement: Figure S3 — Phylogenetic tree reconstruction by RAxML. Reference phylogenetic tree obtained by Maximum Likelihood analysis of the alignment of 16S reference sequences obtained from RDP and selected by similarity, using CD-HIT. Triangle height indicates phylogenetic diversity within each group. On the right hand panel, relative abundances of each taxon. (PDF) [file pone.0074914.s003.pdf]

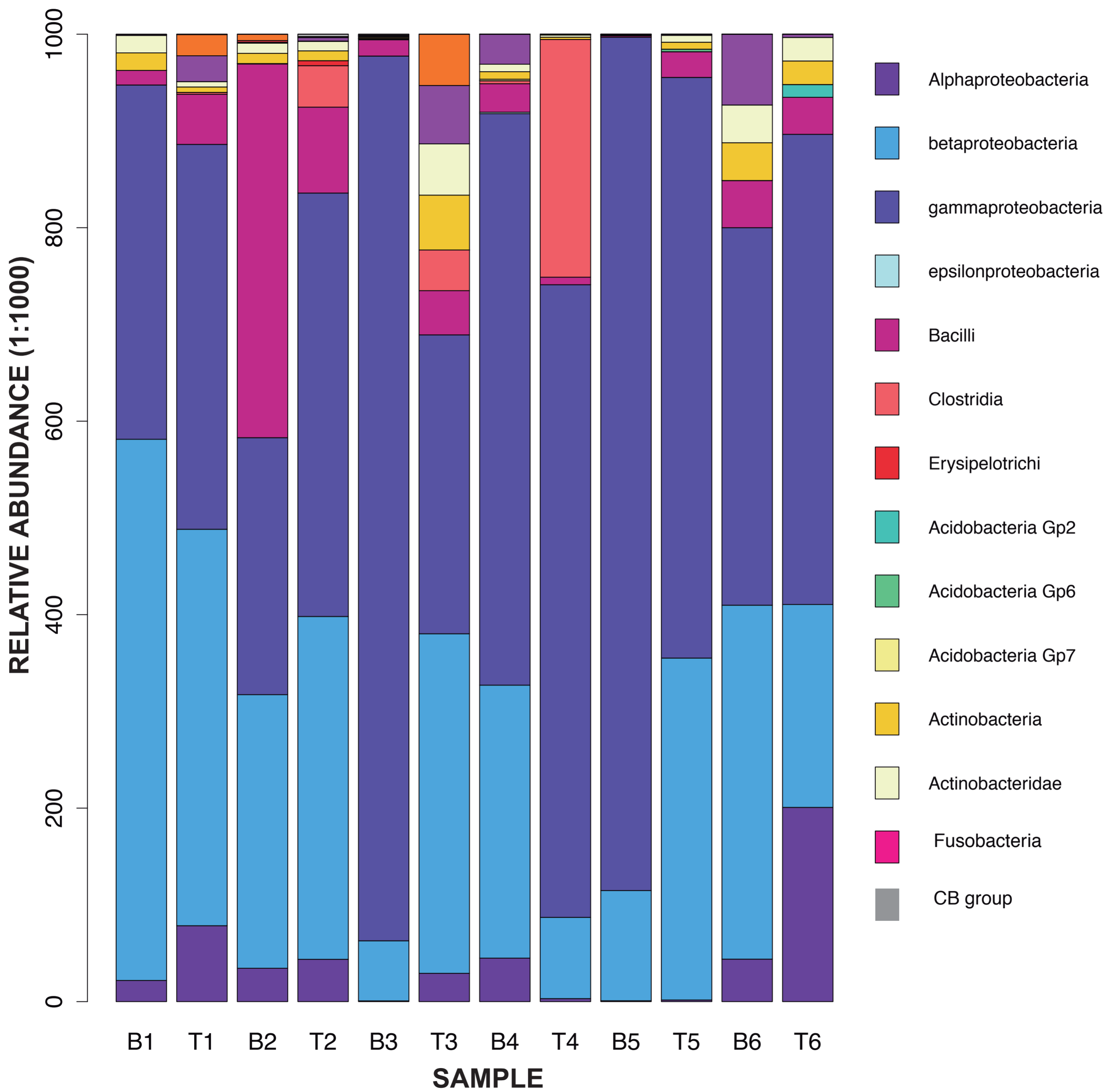

Supplement: Figure S4 — Comparison of the relative abundance of bacteria in skin samples using both methodologies of extraction. 16S DNA sequences were assigned to the genus level. Only genera with more than three sequences assigned were used for the analysis. Then whole information was clustered to class level. Methods are named by the capital letter (B from Bacterial Enrichment extraction and T from Total DNA extraction). (PDF) [file pone.0074914.s004.pdf]

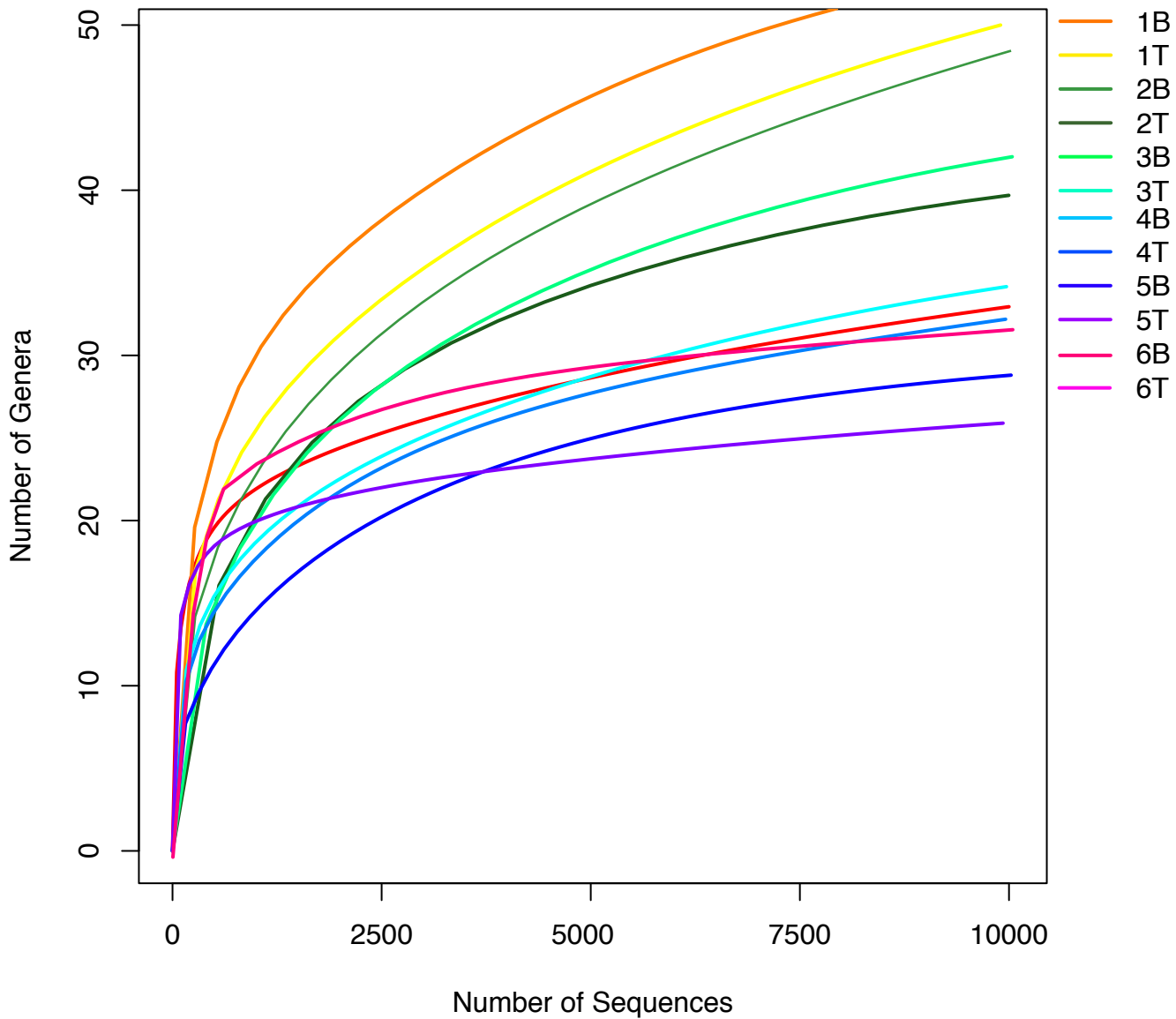

Supplement: Figure S5 — Phylogenetic diversity rarefaction curves for the different samples and methods. Rarefaction curves based on phylogenetic clusters at 97% similarity. Curves were normalized to 10,000 sequences according to the expected slope. (PDF) [file pone.0074914.s005.pdf]

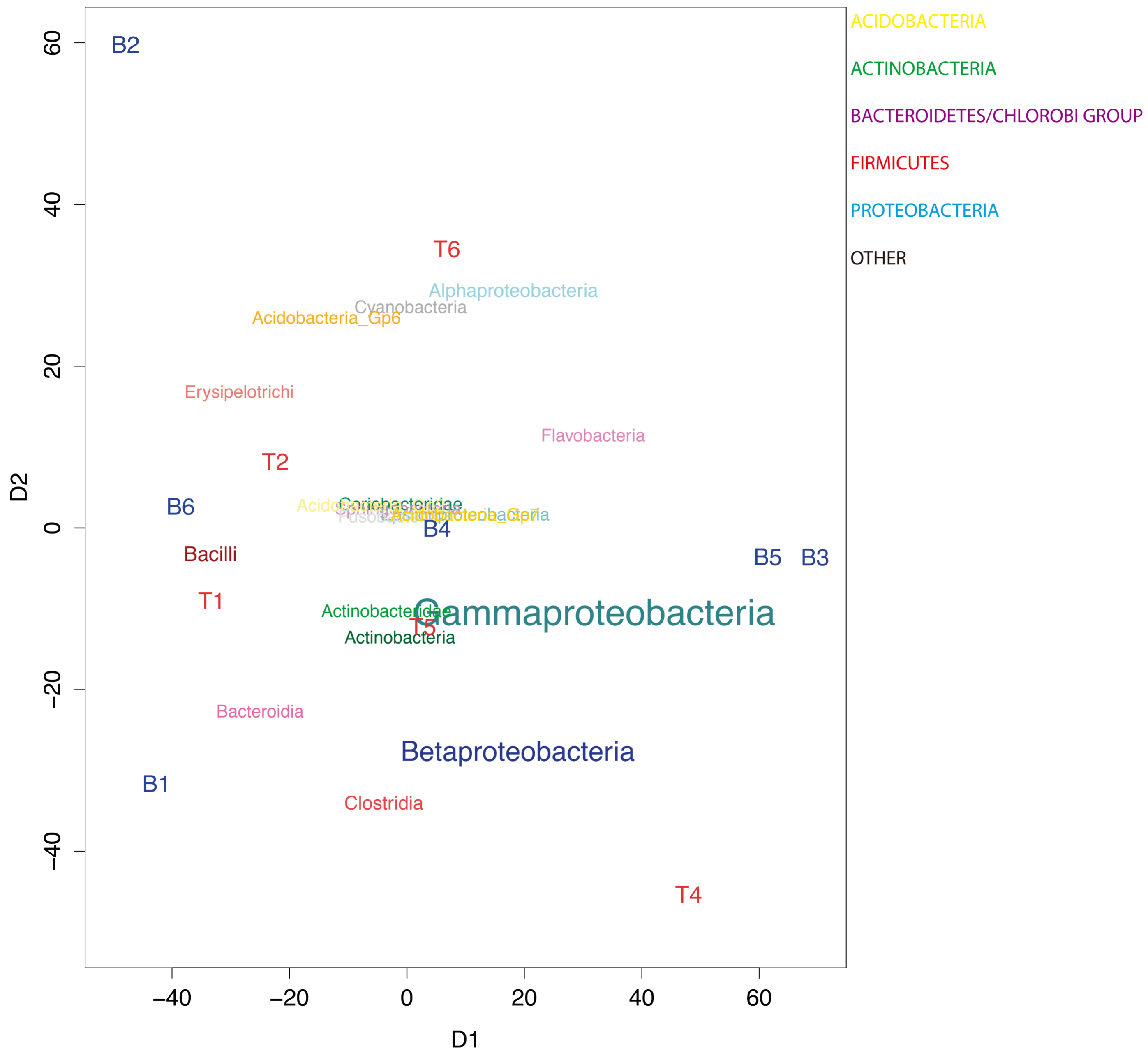

Supplement: Figure S6 — Nonmetric Multidimensional Scaling Analysis of 16S rDNA diversity in standard and bacterial Enrichment methods. First, NMDS was constructed for Sample diversity, using a Manhattan distance matrix and 20 replicates. The resulting rank matrix was reduced to two dimensions, which were used to construct the graph. Colors (blue and red) separate both methods following the same pattern as in figures in the main paper. NMDS matrix was constructed for taxa diversity using the same distance algorithm. Both matrices were normalized one to each other to be comparable. Taxa diversity was plotted by name. Letter size was associated with the relative mean abundance of each taxon. Color was selected by class, using a phylum-based chart. (PDF) [file pone.0074914.s006.pdf]

# Non-metric Multidimensional Scaling analysis of genera relative abundances

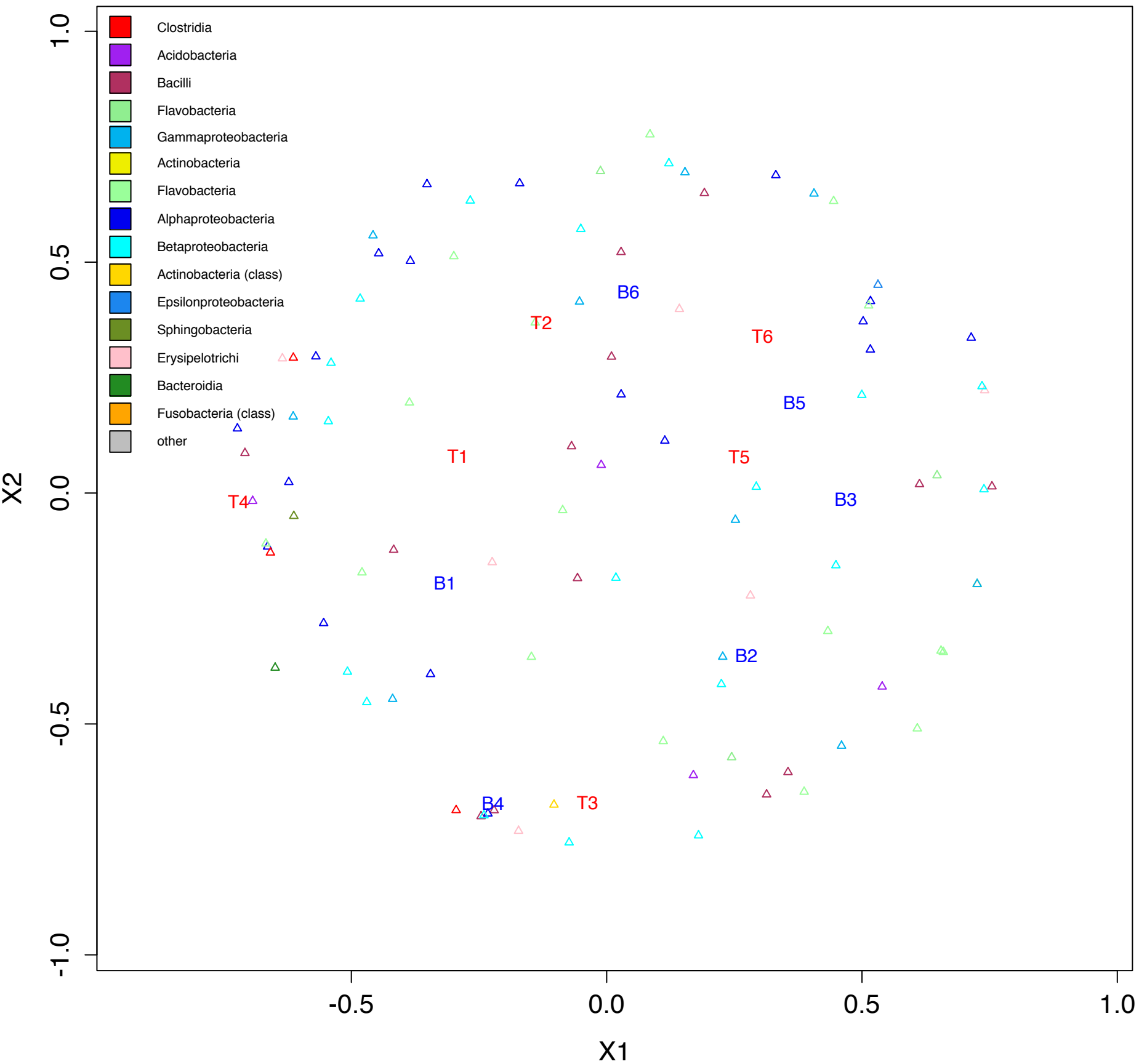

Supplement: Figure S7 — NMDS analysis of 16S rDNA diversity in standard and bacterial enrichment methods (alternative view). Additional perspective of the NMDS taxonomic distribution. Taxonomic categories (genera) are colored according to their class category classification. (PDF) [file pone.0074914.s007.pdf]

# Canonical Correspondence Analysis

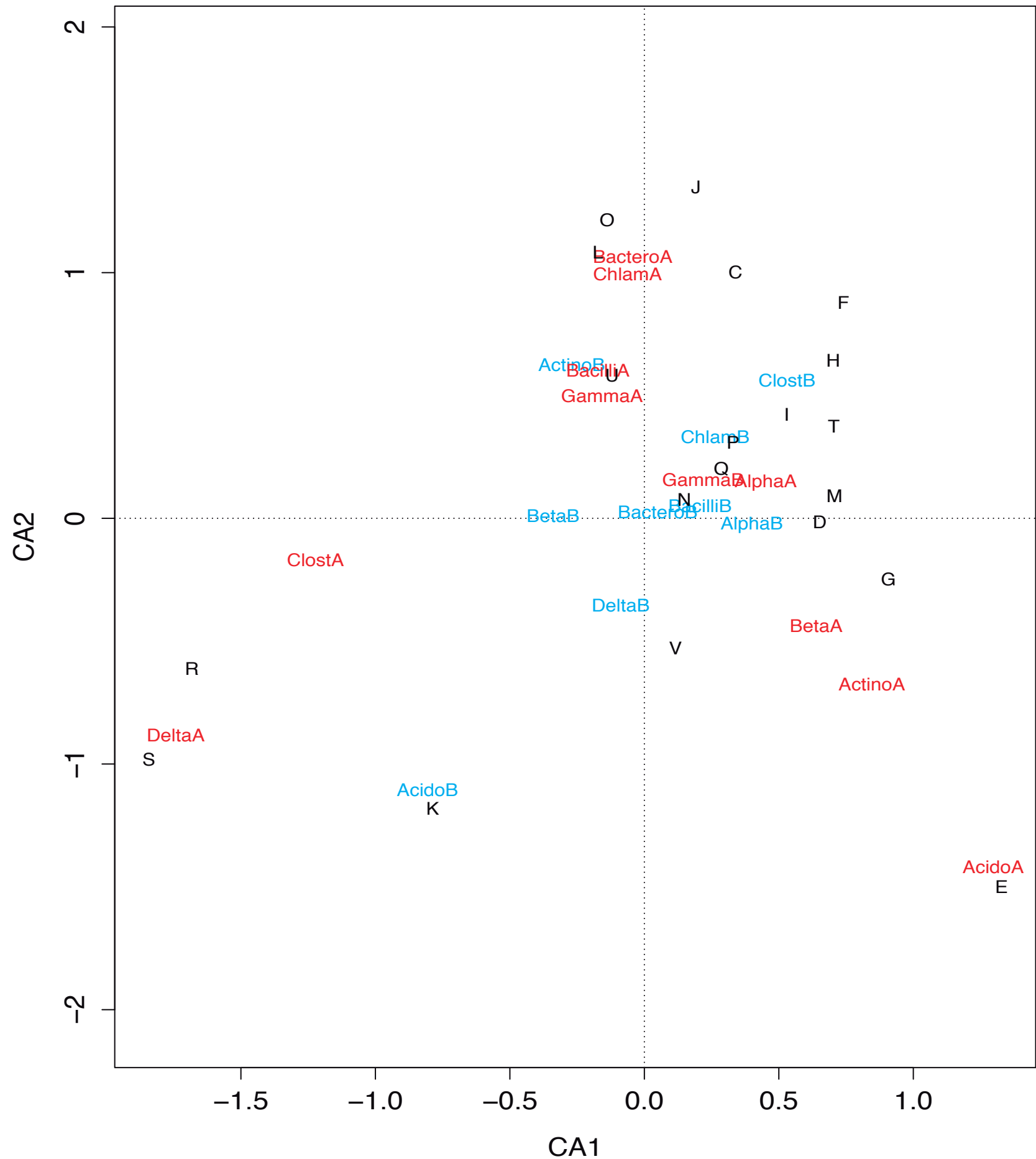

Supplement: Figure S8 — Correspondence Analysis (CoA) of the bacterial function in skin samples, separated by taxa. Samples are represented by color (Sample A in red, sample B in blue), and functions by letter, using the standard eggNOG function category code. (PDF) [file pone.0074914.s008.pdf]

# NMDS function

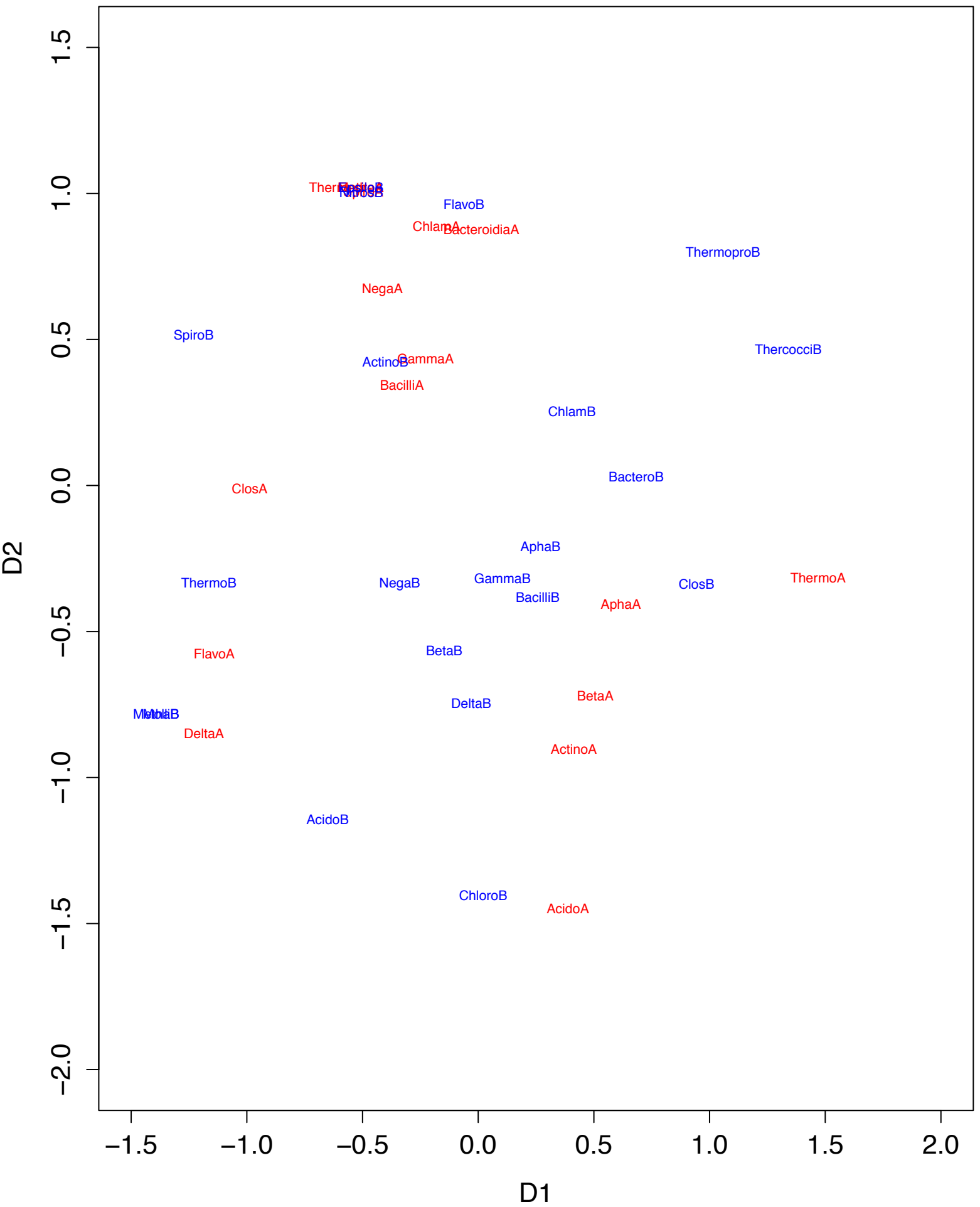

Supplement: Figure S9 — Non-metric Multidimensional Scaling analysis of the taxonomic-based functional diversity for metagenomic samples. Manhattan Distance matrix was constructed for each of the sample-specific taxonomic clusters. Rank classification and dimension scaling was constructed based on the distance matrix. Samples are represented by colour (Sample A in red, Sample B in blue). (PDF) [file pone.0074914.s009.pdf]

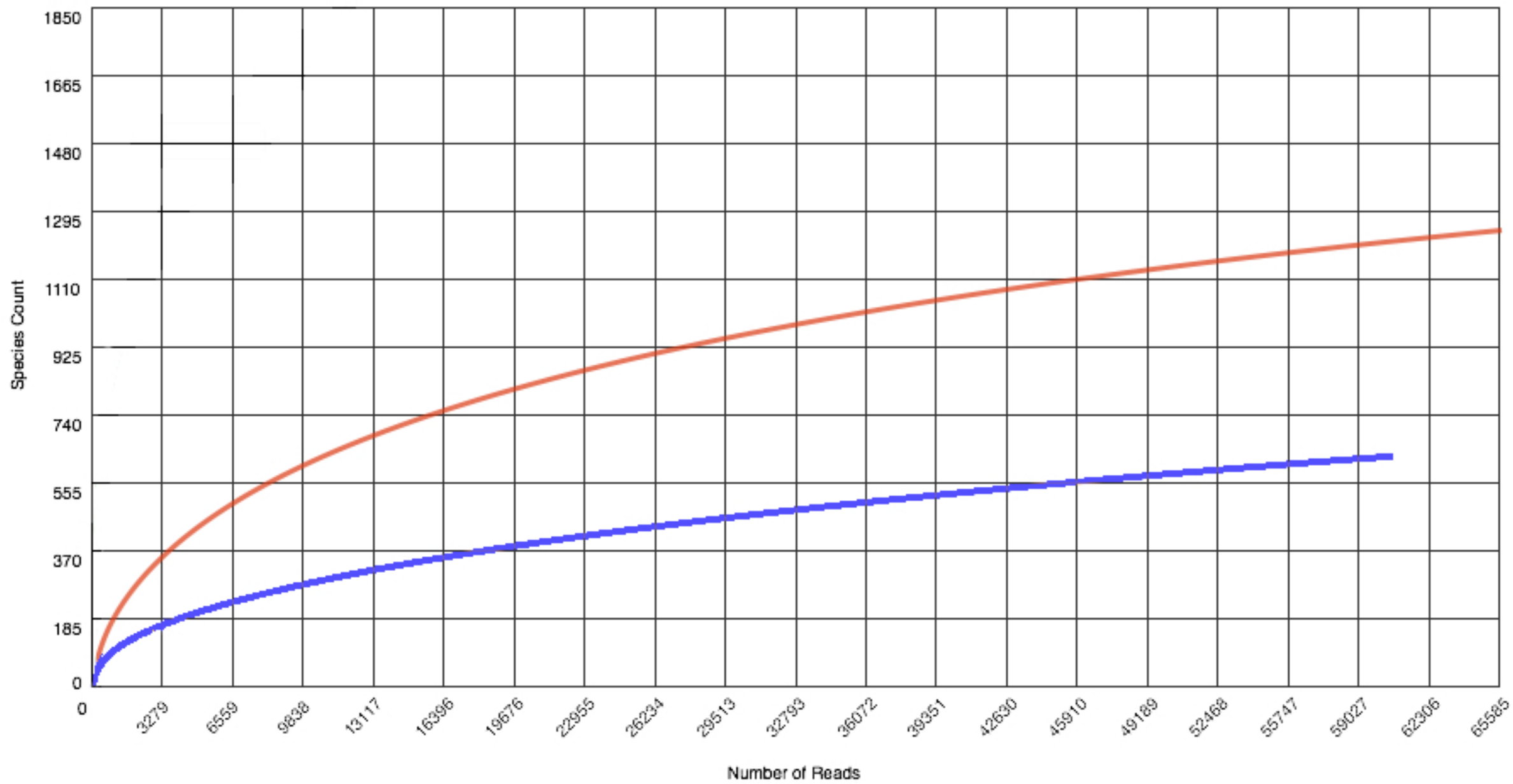

Supplement: Figure S10 — Phylogenetic diversity rarefaction curves for metagenomic samples. Rarefaction curves were constructed by random sampling of reads with taxonomic assignment, using the taxonomic information previously obtained. (PDF) [file pone.0074914.s010.pdf]

# Principal coordinate analysis of genus relative abundances

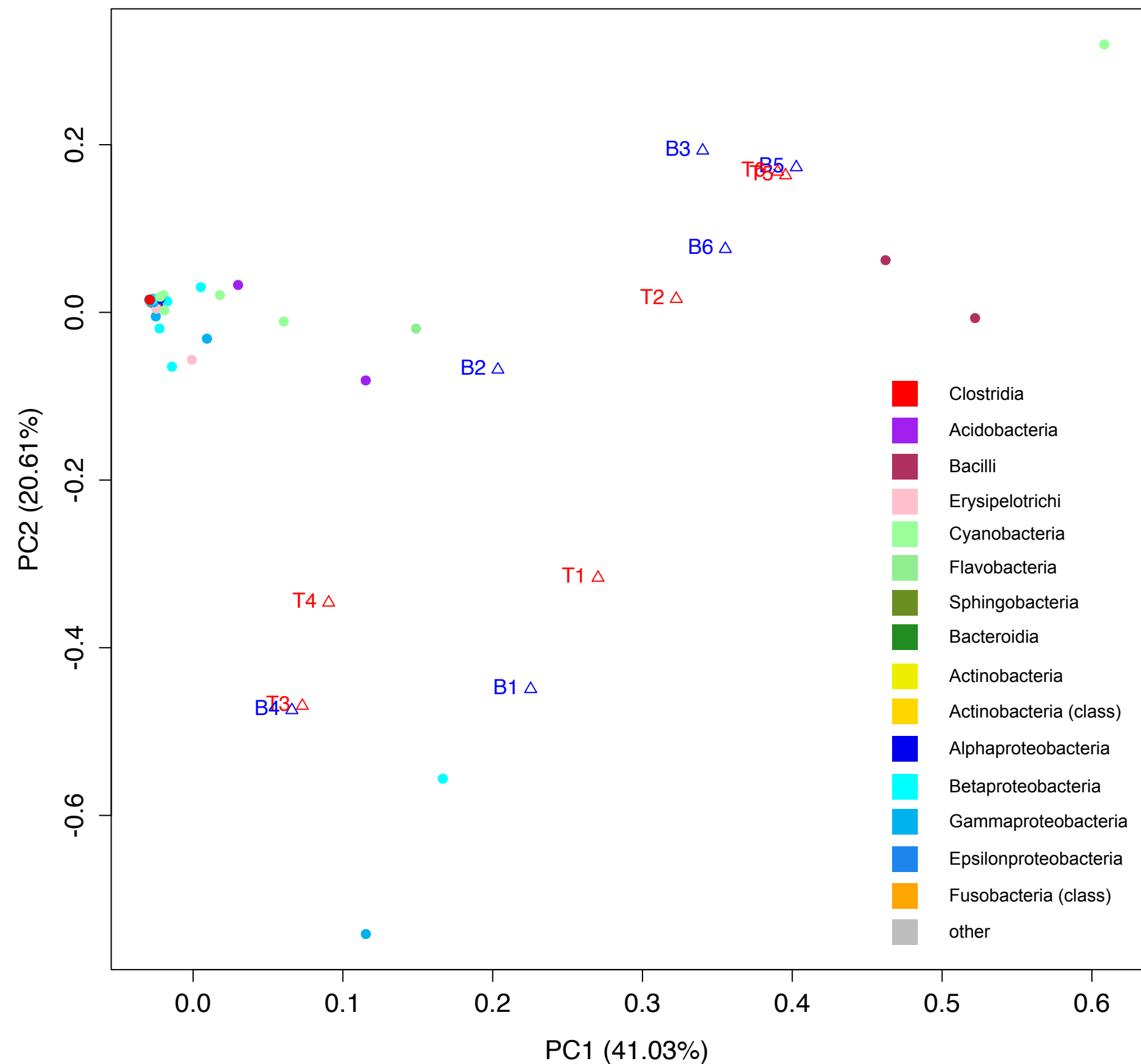

Supplement: Figure S11 — Principal Coordinate Analysis of 16S taxonomic distribution abundances. Bray-Curtis Distance matrix was calculated based on the resampled taxonomic abundances at genus level, for each sample from the validation step. Genus were colored according to their ‘class’ category. Extraction methods were labelled with a capital letter (B as bacterial enrichment and T as total extraction). (PDF) [file pone.0074914.s011.pdf]

# Principal Coordinate Analysis of the taxonomic relative abundances

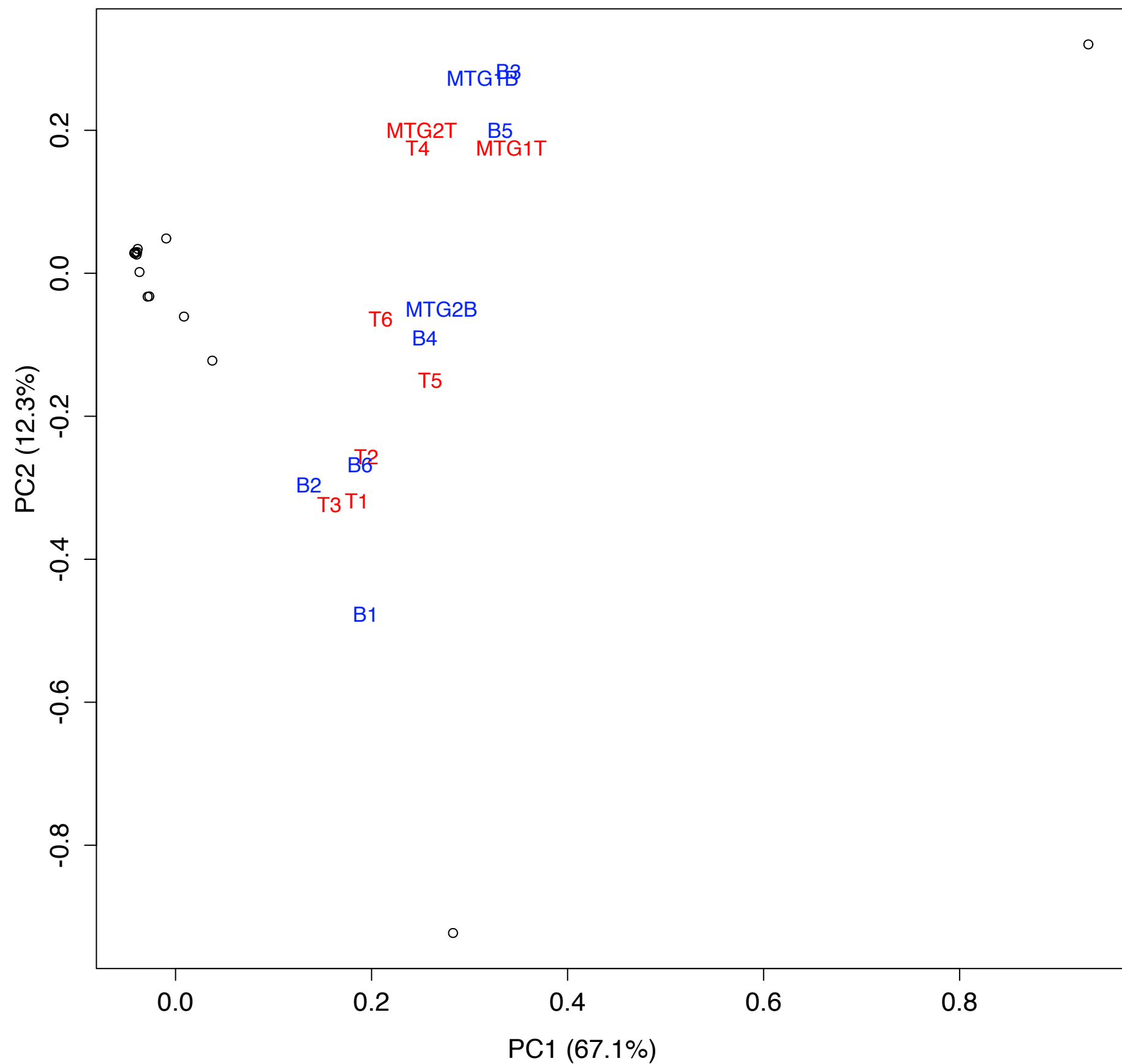

Supplement: Figure S12 — Principal Coordinate Analysis of 16S taxonomic distribution abundances. Samples A and B were processed according to the validation step of 16S rRNA amplification, using both methodologies. The Bray-Curtis Distance matrix was calculated for each sample, and dimension scaling was performed using the PCoA method. Samples were named and highlighted by method (bacterial enrichment method in blue, total DNA extraction in red).. (PDF) [file pone.0074914.s012.pdf]

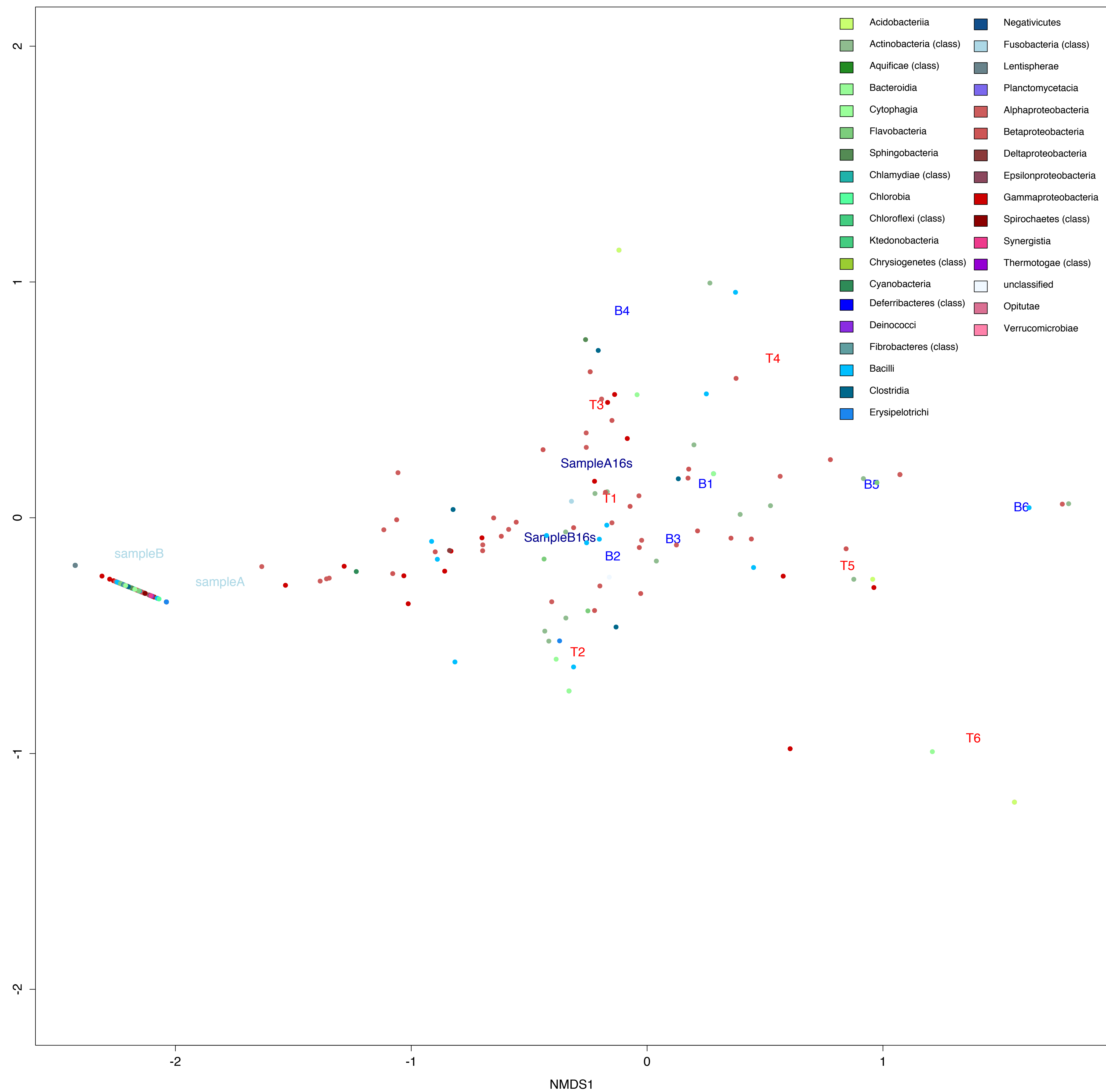

Supplement: Figure S13 — NonMetric Multidimensional Scaling taxonomic analysis of 16S and Metagenomic datasets. The metagenomic method was compared to 16S rRNA amplification in the same samples. In light blue, we see the Samples A and B metagenomic datasets and in dark blue the 16S amplification of the same samples. Color dots show taxonomic entities at genus level, colored by class. As control we have included the 16S rRNA amplifications of the remaining samples included in the work to compare the relative positions on the 2-dimensional space of the metagenomic and the 16S rRNA methodologies. (PDF) [file pone.0074914.s013.pdf]

- Bacterial Enrichment
- Total DNA extraction
- Metagenomic samples

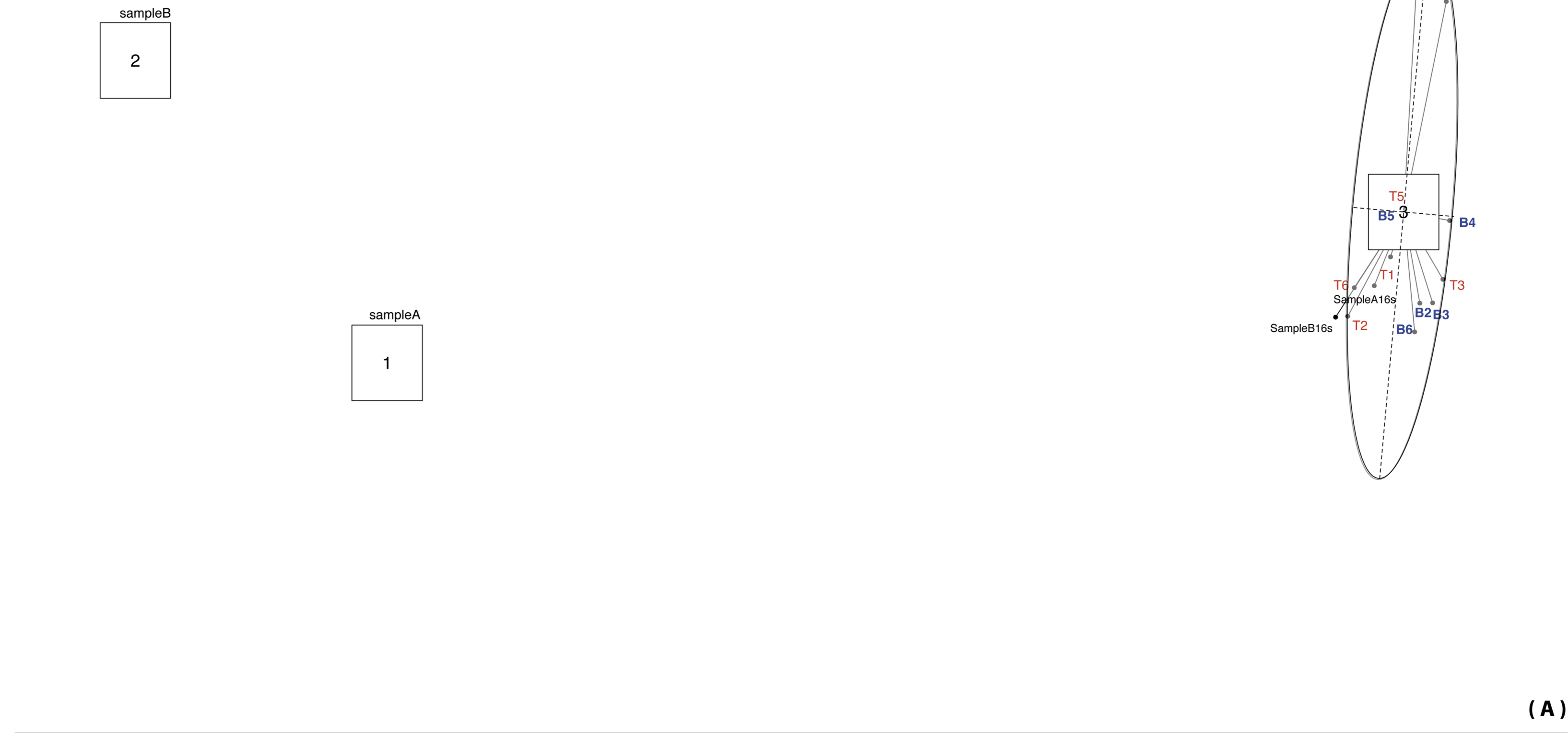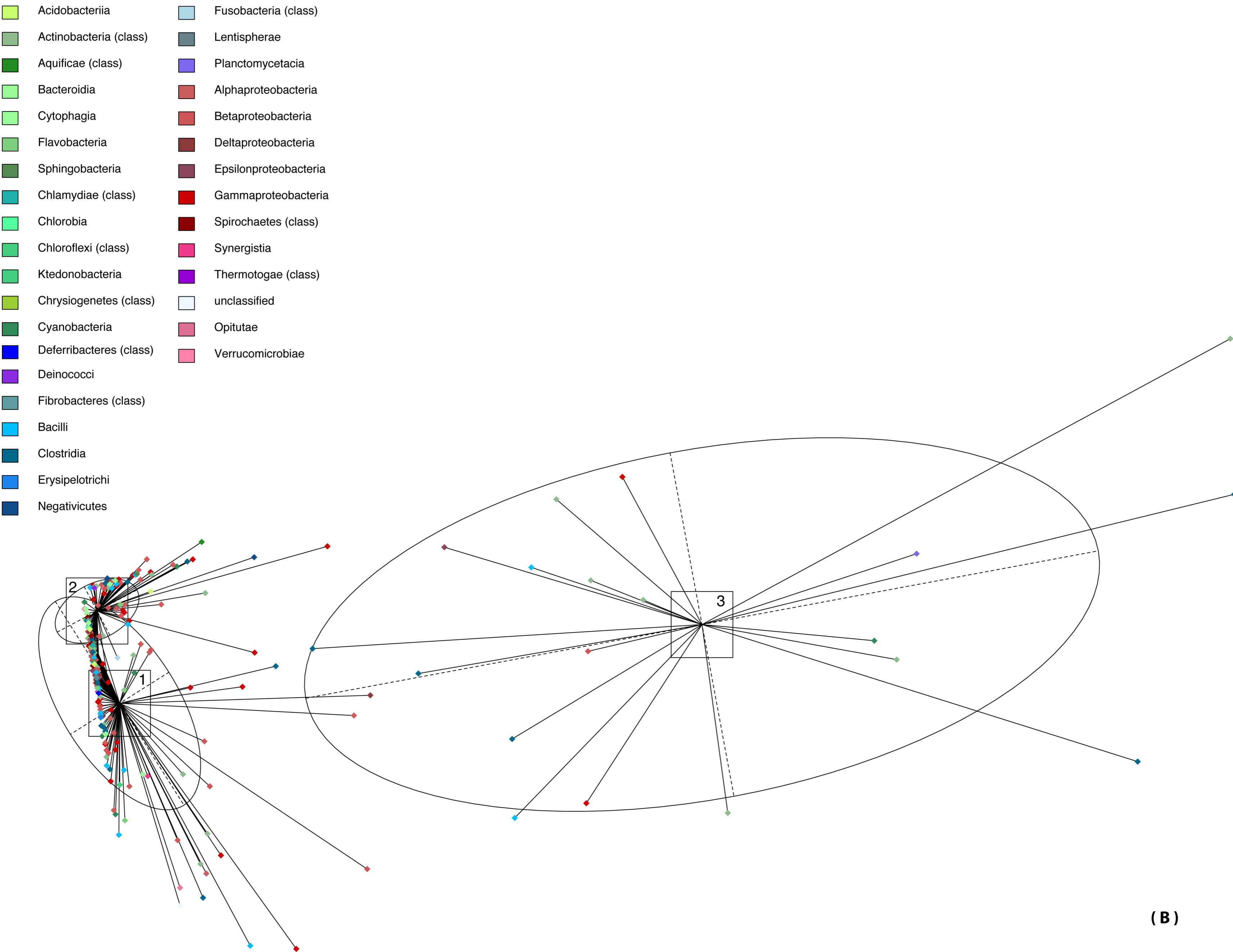

Supplement: Figure S14 — 16S rRNA and metagenomic data comparison and between-class analysis of Taxonomic assignment. A) Principal Coordinate Analysis of Sample dissimilarities. The Bray-Curtis distance was calculated and used to calculate the principal coordinates. Color tags and names were used to differentiate between the three different methodologies: Metagenomic Assignation (in black), bacterial enrichment (blue) and total DNA isolation (red). B) Principal Coordinate Analysis of taxa dissimilarities. Points show the genera distribution across the dimension-reduced space, and color tags were used to differenciate taxonomic units (at class level). To construct the clusters in both cases, the Calinski-Harabasz index was used to estimate the optimal number of clusters. Cluster analysis was performed using the cluster package in R. Between-class analysis was performed to assess the variance from the centroid point for each cluster. Graphical interpretation was performed using the package ade4. (PDF) [file pone.0074914.s014.pdf]
