# Supplementary material for: A New Method for Extracting Skin Microbes Allows Metagenomic Analysis of Whole-Deep Skin
Source: PLoS One. 2013 Sep 20;8(9):e74914. doi: 10.1371/journal.pone.0074914 (PMC3779245; doi:10.1371/journal.pone.0074914)
Supplement: File S1 — Supplementary materials and methods. (DOCX) [file pone.0074914.s015.docx]

**SUPPLEMENTARY MATERIALS AND METHODS**

**Procedure details**

*Skin enzymatic digestion and bacterial first separation*

One skin portion for each individual, 6 mm wide and 5-10 mm deep was incubated in 1.5 mL of buffered enzymatic solution containing sterile HBSS 1X(Invitrogen, Paisley PA4 9RF, UK), collagenase (1 mg/mL) (Sigma-Aldrich. St. Louis, MO), dispase II (1 mg/mL) (Roche Applied Science. Penzberg, Germany), and trypsin 0.025% (Sigma-Aldrich) for 30 min at 37ºC constantly shaking. We included a mock empty sample that followed the same protocol than the rest of samples. The cell saturated solution was then neutralized with BSA 3%-EDTA and stored on ice, and the remaining skin was re-incubated with a new fresh aliquot of enzymatic solution for 30 more minutes. This digestion was repeated until no remaining skin was observed. Three digestions were needed to obtain the total homogenization of the skin sample.

Sterilized nylon filters of 80, 20 and 11 µm (Millipore,Billerica, MA) were preincubated in tween20 (Sigma-Aldrich) 0.1% for 10 min to facilitate the filtration, avoiding the possible adherence of bacteria to the filters, and mounted in a sterile support (swinnex, Millipore). The remaining tween20 was then washed away with BSA 3% in PBS (Sigma Aldrich). The cell suspension was filtered using the filters in a decreasing order, to sequentially reduce the amount of eukaryotic cells in the suspension. To avoid the attachment of the prokaryotic cells to the filters, 10 mL of fresh HBSS was filtered to drag and collect the maximum amount possible of prokaryotes. Cells were washed and concentrated by centrifugation in 500 µL of nuclease-free PBS and quantified by spectrophotometry.

*Bacterial-mitochondrial separation by flow cytometry*

Eukaryotic organelle contamination is an important issue to have in mind in bacterial diversity studies since they may be amplified by universal 16S rRNA primers[1]. Given that the original tissue was a complete slice of skin, and skin is rich in mitochondria, which may be released during the filtration, a cytometric separation was performed using a FACSAria II SORP cell sorter (Becton Dickinson, San Jose, CA), as follows: the remaining cells were centrifuged for 3 min at 12K rpm, treated with 25 µM of DNAse I (NEB.Hitchin, Herts. UK) stock solution (1mg/mL) for 20 min., to remove any possible released DNA, and resuspended in 1 mL of 70% ethanol, leaving the cells at 4ºC overnight to fix. Ethanol was washed away three times with PBS. Propidium Iodide (PI) staining solution was prepared as follows: 50 µL of PI stock solution (1 mg/mL) were diluted together with 50 µL of RNAse A (Qiagen, Valencia. CA) stock solution (both 1 mg/mL) in PBS to a final volume of 1 mL. Cells were resuspended with staining solution and incubated at 4ºC overnight. The cell sorter was prepared according to the type of cells we were trying to separate. Flow cytometry data were collected on the cell sorter using a 85 µm nozzle setup. A single laser assay was carried out using a 488-nm blue laser. Forward (FSC) and Side scatter (SSC) were collected on a logarithmic scale to cover a wide range of particle size detection (<1 to 10 µm) and SSC and PI thresholds were set at 300 and 200 respectively to exclude electronic noise but not low FSC nor low PI emitting particles. PI fluoresecence was measured through a bandpass (BP) 605/40 filter. Acquisition was stopped when 15,000 gated events were collected in the PI vs. autofluorescence dot plot. Autofluorescence was detected in the green channel through 525/30 BP filter. Gates for cell sorting were established on low, medium and bright PI relative fluorescence intensity accordingly to the size and DNA content by three different test controls.

First, sterile inert beads (Sigma Aldrich) of 1, 2.5 and 10 µm of diameter were used to locate the specific size window for bacteria and mitochondria. A size-specific gate was defined. Two different gates were defined for the mitochondria (1-2.5 µm) and bacteria (1-10 µm) sizes. Given that beads do not fluoresce, PI background was set using the same beads. Second, *Escherichia coli* cells from a pure culture were used as bacterial standard to assess size and genomic content. Finally, bacteria from feces, which are almost free of eukaryotic cells[2] were used as a standard complex community to take into account possible deviations on the size range (see below for more information on the control preparation). Controls were sorted sequentially, and all variables (Forward scatter, side scatter, fluorescence range, autofluorescence) were set for further analyses. Isolated cells were then sorted using the previously set thresholds, removing all out-of-threshold events. Given that the genome size of mouse mitochondria is 16 kb, and accounting for ~ 25X polyploidy, genomic size threshold was set at 1 Mb. All particles containing more than 0.5 Mb of DNA and with a size of 1-10 µm were recovered. Additionally, any particle below the size range of 1µm was recovered.

The remaining cell suspension was centrifuged for 3 min at 8K rpm, to precipitate the cells, and washed with PBS 1X (Ambion. Life Technologies. Austin TX), to eliminate possible eukaryotic debris. The remaining cells were then incubated in a preparation of buffer solution (PBS 1X) and lysozyme (10 mg/mL) for 30 min at 37°C, and subsequently cooled on ice. DNA extraction was performed using a phenol-chloroform standard method. The exact details for DNA extraction are explained below.

Extracted DNA was eluted in 50 µL of nuclease-free Tris-EDTA buffer (TE), and the resulting suspension was quantified using Nanodrop.

As an alternative control protocol, frozen skin samples of similar weight were homogenized in PBS using a mechanical homogenizer (IKA® Ultraturrax. Thermo Fischer), and followed by the standard protocol. Whole genomic DNA was extracted using the same protocol. All the following steps were carried out in parallel for both extraction methods.

**Method validation**

We controlled for presence of bacterial, host, and human DNA in our enriched samples. To verify the presence or absence of bacterial DNA, a standard PCR amplification of the 16S rRNA gene was performed from the purified genomic DNA using the universal primers 8F (5'-AGAGTTTGATCCTGGCTCAG-3') and 355R (5'-CTGCTGCCTCCCGTAGGAGT-3') [3]. For each 50 μL reaction, conditions were as follows: 5.0 μL of 10X buffer with MgCl2 (Roche Applied Science), 2 μL of dNTP mix (10 mM each; Roche Applied Science), 1.5 μL of each primer (20 μM; IDT), 5 μL of DMSO, 1.5ng of bacterial genomic DNA, and 1 U of FastStart High Fidelity Taq Polymerase (Roche Applied Science). Thermocycling conditions were the following: initial denaturation at 95°C for 2 min, followed by 30-32 cycles of a 30-sec 95°C denaturation, 30-sec annealing at 55°C, 1-min elongation at 72°C, and a final extension of 8 min at 72°C. PCR products were visualized on a 1% agarose gel. All of them should contain just one band corresponding to 350 bp. Negative and positive control PCR reactions were performed with each set of amplifications and in all cases did produce the expected result.

Host DNA was detected by means of the rodent-specific IRGA6 gene [4,5], which was PCR-amplified with primers 269F (5'-GAGGCATTGGGAATGAAGAA-3') and 668R (5'-GCAATGCCATTCTCCCTAAA-3') following the same conditions than for 16S rRNA gene amplification. Experimental contamination (which may alter the bacterial composition by introducing exogenous prokaryotes) was measured by detecting human DNA; to that effect, we amplified the NPIP gene[6], which is specific for the primate lineage and is not shared with rodents. This combination of IRGA6 and NPIP genes allows us to discriminate between host and experimental contamination.

To assess the actual ratio between bacterial and mouse DNA isolated, a qPCR experiment was performed. First, the 16S rRNA gene V1-V2 region was amplified using primers 63F (5'-GCAGGCCTAACACATGCAAGTC-3') and 355R (5Ј CTGCTGCCTCCCGTAGGAGT-3')[7,8]. The possible host DNA was also quantified using the IRGA6 gene primers 151F(5' -AGAGCACACCGAGGGCTATTC-3') and 257R (5'-GAACAGCTGACCCATGACTTCA5'). To perform an absolute quantification and assess the efficiency of the PCR, a standard curve was constructed by amplifying serial dilutions of known quantities of *E. coli* DNA and mouse DNA for each one. The qPCR experiment was performed on a LightCycler 480 (Roche Applied Science) using optical grade 385-well plates. Each 10 μL reaction included 5 μL SybrGreen Master Mix (Roche Applied Science), 1 μL each primer (10 μM), 1 μL water, and 2 μL of DNA. For each DNA sample, three replicates were performed. The cycling conditions used were as follows: initial denaturation at 95°C for 4 min, followed by of 40 cycles of 10 s 95º denaturation and 60-sec 60ºC elongation. The standard curve equations were: C_q_ =-0.43x + 8.43; for *E. coli* and C_q_=-0.46x+13.54 for IRGA6, both with R²=1 and an efficiency of 2.

According to the amount of DNA obtained after the protocol, the following nested PCR protocol was performed: for each sample we amplified 16S rRNA genes using 8F and 1510R (5'-CGGTTACCTTGTTACGACTT-3') covering the nine variable regions without any phylogenetic bias[9]. The mix conditions were the same than in the previous amplification, and the cycling conditions were as follows: a first denaturation step of 2 min at 95ºC, followed by 25 cycles of 30 s at 95ºC, 30 sat 55ºC and 90 s at 72ºC, followed by a final extension step of 8 min at 72ºC. This first amplification was followed with a semi-nested PCR using primers 8F and 355R, modified according to Costello et al. [10]. The forward primer (5’-CGTATCGCCTCCCTCGCGCCATCAG-XXXXXXXXXXX-AGAGTTTGATYMTGGCTCAG -3’) that contained the 454 Life Sciences Titanium primer A sequence, followed by a 10 nt error correcting barcode, and the degenerated primer 8F. The reverse primer (5'- CTATGCGCCTTGCCAGCCCGCTCAGTGCTGCCTCCCGTAGGAGT -3') contained the 454 Life Sciences primer B sequence, the broadly conserved bacterial primer 355R, and a two-base linker sequence (‘TC’). All forward and reverse primers were digitally tested using GeneRunner. PCR conditions were modified from the previous amplification step reducing the elongation time to 30 s, and the annealing temperature to 52ºC to allow maximum adherence. Amplification was repeated three times per sample. Replicate amplicons were pooled and visualized on 1.0% agarose gels using Red Safe DNA gel stain in 0.5X TBE. Amplicons were cleaned using the NucleoFast 96-well plates according to manufacturer's instructions.

**Amplicon quantification, pooling, and pyrosequencing.**

Amplicon PCR concentrations were determined using a Nanodrop Spectophotometer (Thermo Fisher, Waltham, MA). Cleaned amplicons were pooled in equimolar ratios into a single tube to a final amount of 700 ng of DNA. Pyrosequencing was carried out using primer A on a 454 Life Sciences Genome Sequencer FLX instrument (Roche Applied Science), to an expected final amount of 5,000 sequences per sample.

**Mouse metagenomic library preparation and sequencing.**

Two independent mouse skin samples were processed to assess the proper ability of this method to perform efficient cheaper metagenomic libraries. Extracted DNA was placed in low-binding tubes and sonicated with a Bioruptor®(Diagenode, Denville, NJ), according to manufacturer’s instructions to obtain a 400-800 bp fragment range. As the amount of DNA was relatively low, fragment distribution was verified using an *E. coli* genomic DNA extraction, processed following the same conditions. Fragmented DNA was then purified using NucleoFast 96-well plates according to manufacturer's instructions, and resuspended on 50µL of TE. Subsequent purification, blunt-end repair, adapter ligation, amplicon quantification and pyrosequencing library generation were carried out following a previously published protocol [11] to avoid a titration step and allowing to work with just 1ng of fragmented DNA. qPCR was performed, as in Zheng Z. et al. [11]] to quantify the exact number of molecules in our samples. Further steps were performed according to the standard Roche protocol.

To further validate the method, samples A and B were re-extracted using both methodologies and 16S rRNA was amplified in both cases to proper compare the three (bacterial enrichment plus 16S rRNA amplification, bacterial enrichment plus metagenomic sequencing and taxonomic characterization, and total DNA extraction and 16S rRNA amplification) possibilities.

**Amplification analysis**

Amplicons were processed and analyzed following the procedure described previously [12]. First, only sequences between 200 and 400 nt with a quality score >25, containing unambiguous characters, and that did not contain an uncorrectable bar-code, were used. The remaining sequences were assigned to samples by examining the 10-nt bar-code. Pyrosequencing noise and chimera sequences were filtered using Ampliconnoise, implemented in qiime[13] and Chimera Slayer[[14]. Similar sequences were clustered into operational taxonomic units (OTUs, also called phylotypes) using CD-HIT[15] with a minimum coverage of 99% and a minimum identity of 98%. For each OTU, the longest sequence was chosen as representative and was aligned using BLAST[16] against the “nt” database and against a local, self-formated RDP database[17-19]. Only hits with a coverage over 99% and an E-value below 10^-10^ were kept. Homopolymeric regions were corrected using the reference sequence (GI) of the top result. In case of taxon ambiguity, the read was assigned to the lowest common ancestor in the NCBI reference taxonomy. OTUs defined as environmental undefined sequence, unknown, or assigned to a level below family were removed from the analysis. A maximum likelihood (ML) phylogenetic tree was constructed using RaxML [20] using 100 bootstrap generations and the default configuration. Setting the resulting probabilities as prior probabilities and the maximum likelihood tree as a seed tree, we calculated the posterior probabilities and the most plausible tree shape using phyloBayes[21] with the default configuration.

Shannon, Simpson (1-D) diversity indexes and Chao richness index were calculated for both samples, using the vegan R-package[22]. Multidimensional methods, Correspondence analysis (CA), Canonical Correspondence Analysis (CCA), principal coordinate analysis (PCoA) and Non-metric multidimensional scaling (NMDS) were performed using the *vegan* and the *ecodist* R packages[23]. To test dissimilarities between samples, within samples, and between methodologies, the *adonis* function of the *vegan* package was used, with at least 200 initial configuration permutations.

**Metagenomic sequences post-processing and analysis**.

Shotgun sequences were processed as follows. First, reads from the host were eliminated using Deconseq[24]. Filtered datasets were then uploaded to MG-RAST[25] in FastQ format and were deposited in the MG-RAST database[26] with the accesion numbers 4496968.3 and 4496969.3. Uploaded reads were filtered using the quality control (QC) pipeline implemented in MG-RAST[27]. Reads with ambiguities, low complexity, or short size were eliminated from the analysis. Artificial duplicated sequences were filtered[27].

Taxonomical and functional abundances were calculated with MG-RAST using data normalization and the best representative method. Original files were split in taxonomic specific files to assess functional differences among taxa using phymmBL[28]. At the same time, COG and eggNOG alignments were downloaded and specific Hidden Markov Models (HMM) were constructed for each alignment using HMMer 3.0[29]. Taxonomy-specific reads were aligned using the HMMSearch from HMMer. Results were joined in a single file for each taxon-specific file, and parsed using a customized script written in Perl. Functional assignation was performed according to the highest likelihood obtained. Reads with likelihoods below 10^3^ were discarded and considered as “unclassified”. Comparisons and NMDS were performed using the R package *ecodist*.

**Standard Extraction method.**

One skin portion for each individual, 6mm wide and 5-10mm deep was placed in a flat well 2mL tube. Tissue was homogenized with a mechanical homogenizer IKA Ultraturrax (Thermo Scientific, Waltham, MA). 300µL of tissue lysis buffer (100mM Tris-HCl pH 8.0, 1mM EDTA pH 7.6, 100mM NaCl, SDS 10%) and 50µL of Proteinase K (Roche Applied Science. Penzberg. Germany) were added to the homogenized tissue, and mix was incubated for 30 min at 56ºC. Homogenized tissue was ice cooled, and 300µL of Lysozyme (10mg/mL, Sigma-Aldrich, Munich, Germany) buffer in nuclease-free PBS (Ambion, Paisley, UK) was added. The mix was incubated for 30 min at 37ºC. 600µL of Phenol:Chlorophorm:Isoamilalcohol (25:24:1) were added, mixed, and spinned at maximum speed for 3 minutes to separate the organic and the aquose phase. The aquose phase was placed in a new tube. The phenol-chlorophorm separation was repeated twice, the last time using only chlorophorm. Around 400µL of aquose phase were recovered. 800µL of absolute ethanol and 40µL of ammonium acetate (5M) were added to the aquose phase. The solution was mixed and incubated at -80ºC for 2h. DNA was precipitated and washed with 70%ethanol, air dried, and resuspended on 50µL of nuclease-free distilled water.

**Standard feces microbiota isolation and PI staining**

10g of feces were collected from a healthy volunteer on a sterile collection 10mL tube with 15mL of PBS. Feces were homogenized and centrifuged for 8 minutes at 4K rpm. Bacteria precipitated as a whitish layer over the fecal debris. Bacteria was then resuspended, using a pipette, and the whole volume was recollected, and split in 10 independent eppendorf tubes. Bacteria were centrifuged for 3min at 8K rpm and resuspended in 70%ethanol in a single eppendorf tube. Bacteria were resuspended and incubated O/N at 4ºC to fix. Ethanol was washed away three times with PBS. 100µL of propidium iodide (PI) staining solution with RNAse was used to stain the bacteria O/N in black eppendorf tubes to block light. This bacterial suspension was used afterwards as bacterial DNA staining and size control.

**METHODOLOGICAL REFERENCES**

1. Benítez-Páez A, Álvarez M, Belda-Ferre P, Rubido S, Mira A, et al. (2013) Detection of Transient Bacteraemia following Dental Extractions by 16S rDNA Pyrosequencing: A Pilot Study. PLoS ONE 8: e57782. doi:10.1371/journal.pone.0057782.g004.

2. Arumugam M, Raes J, Pelletier E, Le Paslier D, Yamada T, et al. (2011) Enterotypes of the human gut microbiome. Nature 473: 174–180. doi:10.1038/nature09944.

3. Baker GC, Smith JJ, Cowan DA (2003) Review and re-analysis of domain-specific 16S primers. Journal of Microbiological Methods 55: 541–555. Available: <http://eutils.ncbi.nlm.nih.gov/entrez/eutils/elink.fcgi?dbfrom=pubmed&id=14607398&retmode=ref&cmd=prlinks>.

4. Bekpen C, Hunn JP, Rohde C, Parvanova I, Guethlein L, et al. (2005) The interferon-inducible p47 (IRG) GTPases in vertebrates: loss of the cell autonomous resistance mechanism in the human lineage. Genome Biol 6: R92. doi:10.1186/gb-2005-6-11-r92.

5. Bekpen C, Marques-Bonet T, Alkan C, Antonacci F, Leogrande MB, et al. (2009) Death and Resurrection of the Human IRGM Gene. PLoS Genet 5: e1000403. doi:10.1371/journal.pgen.1000403.s007.

6. Johnson ME, Viggiano L, Bailey JA, Abdul-Rauf M, Goodwin G, et al. (2001) Positive selection of a gene family during the emergence of humans and African apes. Nature 413: 514–519. doi:10.1038/35097067.

7. Castillo M, Martín-Orúe SM, Manzanilla EG, Badiola I, Martín M, et al. (2006) Quantification of total bacteria, enterobacteria and lactobacilli populations in pig digesta by real-time PCR. Vet Microbiol 114: 165–170. Available: <http://linkinghub.elsevier.com/retrieve/pii/S0378113505004414>.

8. Grice EA, Kong HH, Renaud G, Young AC, NISC Comparative Sequencing Program, et al. (2008) A diversity profile of the human skin microbiota. Genome Research 18: 1043–1050. doi:10.1101/gr.075549.107.

9. Schloss PD, Gevers D, Westcott SL (2011) Reducing the effects of PCR amplification and sequencing artifacts on 16S rRNA-based studies. PLoS ONE 6: e27310. doi:10.1371/journal.pone.0027310.t004.

10. Costello EK, Lauber CL, Hamady M, Fierer N, Gordon JI, et al. (2009) Bacterial Community Variation in Human Body Habitats Across Space and Time. Science 326: 1694–1697. doi:10.1126/science.1177486.

11. Zheng Z, Advani A, Melefors Ö, Glavas S, Nordström H, et al. (2011) Titration-free 454 sequencing using Y adapters. Nat Protoc 6: 1367–1376. doi:10.1038/nprot.2011.369.

12. Garcia-Garcerà M, Coscollá M, Garcia-Etxebarria K, Martín-Caballero J, González-Candelas F, et al. (2012) Staphylococcus prevails in the skin microbiota of long-term immunodeficient mice. Environmental Microbiology. doi:10.1111/j.1462-2920.2012.02756.x.

13. Caporaso JG, Kuczynski J, Stombaugh J, Bittinger K, Bushman FD, et al. (2010) QIIME allows analysis of high-throughput community sequencing data. Nat Meth 7: 335–336. doi:10.1038/nmeth0510-335.

14. Haas BJ, Gevers D, Earl AM, Feldgarden M, Ward DV, et al. (2011) Chimeric 16S rRNA sequence formation and detection in Sanger and 454-pyrosequenced PCR amplicons. Genome Research 21: 494–504. doi:10.1101/gr.112730.110.

15. Li W, Godzik A (2006) Cd-hit: a fast program for clustering and comparing large sets of protein or nucleotide sequences. Bioinformatics 22: 1658–1659. doi:10.1093/bioinformatics/btl158.

16. Altschul SF, Gish W, Miller W, Myers EW, Lipman DJ (1990) Basic local alignment search tool. J Mol Biol 215: 403–410.

17. Maidak BL, Cole JR, Lilburn TG, Parker CT, Saxman PR, et al. (2001) The RDP-II (Ribosomal Database Project). Nucleic Acids Research 29: 173–174.

18. Cole JR, Chai B, Farris RJ, Wang Q, Kulam-Syed-Mohideen AS, et al. (2007) The ribosomal database project (RDP-II): introducing myRDP space and quality controlled public data. Nucleic Acids Research 35: D169–D172. doi:10.1093/nar/gkl889.

19. Cole JR, Wang Q, Cardenas E, Fish J, Chai B, et al. (2009) The Ribosomal Database Project: improved alignments and new tools for rRNA analysis. Nucleic Acids Research 37: D141–D145. doi:10.1093/nar/gkn879.

20. Stamatakis A, Ludwig T, Meier H (2005) RAxML-III: a fast program for maximum likelihood-based inference of large phylogenetic trees. Bioinformatics (Oxford, England) 21: 456–463. doi:10.1093/bioinformatics/bti191.

21. Lartillot N, Philippe H (2004) A Bayesian mixture model for across-site heterogeneities in the amino-acid replacement process. Mol Biol Evol 21: 1095–1109. doi:10.1093/molbev/msh112.

22. Oksanen J, Blanchet FG, Kindt R, Legendre P, O'Hara RB, et al. (2011) Vegan: community ecology package. R package version 117-10. Available: <http://CRAN.R-project.org/package=vegan>.

23. Urban DL, Goslee SC (2007) The ecodist Package for Dissimilarity-based Analysis of Ecological Data. Journal of Statistical Software.

24. Schmieder R, Edwards R (2011) Fast Identification and Removal of Sequence Contamination from Genomic and Metagenomic Datasets. PLoS ONE 6: e17288. doi:10.1371/journal.pone.0017288.t002.

25. Thomas T, Gilbert J, Meyer F (2012) Metagenomics - a guide from sampling to data analysis. Microbial Informatics and Experimentation 2: 3. doi:10.1186/2042-5783-2-3.

26. Meyer F, Paarmann D, D'Souza M, Olson R, Glass EM, et al. (2008) The metagenomics RAST server - a public resource for the automatic phylogenetic and functional analysis of metagenomes. BMC Bioinformatics 9: 386. doi:10.1186/1471-2105-9-386.

27. Gomez-Alvarez V, Teal TK, Schmidt TM (2009) Systematic artifacts in metagenomes from complex microbial communities. ISME J 3: 1314–1317. doi:10.1038/ismej.2009.72.

28. Brady A, Salzberg SL (2009) Phymm and PhymmBL: metagenomic phylogenetic classification with interpolated Markov models. Nat Meth 6: 673–676. doi:10.1038/nmeth.1358.

29. Madera M, Gough J (2002) A comparison of profile hidden Markov model procedures for remote homology detection. Nucleic Acids Research 30: 4321–4328.
