# Supplementary material for: A New Method for Extracting Skin Microbes Allows Metagenomic Analysis of Whole-Deep Skin
Source: PLoS One. 2013 Sep 20;8(9):e74914. doi: 10.1371/journal.pone.0074914 (PMC3779245; doi:10.1371/journal.pone.0074914)
Supplement: Table S1 — qPCR values for bacterial 16S rRNA and the murine IRGA6 gene. B, samples enriched for bacteria; T, total extraction samples. (DOCX) [file pone.0074914.s016.docx]

| **Sample** | **16S rRNA B** | **16S rRNA T** | **IRGA6 B** | **IRGA6 T** |
| --- | --- | --- | --- | --- |
| 1 | 26.57 | 23.97 | 35.57 | 28.64 |
| 2 | 27.54 | 23.21 | 37.56 | 21.79 |
| 3 | 18.03 | 23.44 | 36.81 | 21.54 |
| 4 | 23.99 | 23.17 | 35.96 | 26.04 |
| 5 | 25.66 | 23.51 | 38.68 | 23.11 |
| 6 | 27.03 | 23.34 | 35.72 | 21.84 |
| mock | 33.08 | 33.46 | 37.27 | 36.89 |
| negative | 32.17 | | 37.92 | |
