# Supplementary material for: A New Method for Extracting Skin Microbes Allows Metagenomic Analysis of Whole-Deep Skin
Source: PLoS One. 2013 Sep 20;8(9):e74914. doi: 10.1371/journal.pone.0074914 (PMC3779245; doi:10.1371/journal.pone.0074914)
Supplement: Table S2 — Diversity measurements of 16S rRNA sequences. Diversity indexes (Shannon diversity index, Chao1 Richness and ACE) were calculated for each sample given a family-based abundance table. B, bacterial enrichment samples; T, total extraction samples; Samples A and B for metagenomic samples. SE, Standard Error; ACE, Abundance-base Coverage Estimator. Undefined (NaN) values appear when all rare taxa are only assigned as singletons. (DOCX) [file pone.0074914.s017.docx]

|  | **N** | **Shannon** | **Chao1** | **SE.Chao1** | **ACE** | **SE.ACE** |
| --- | --- | --- | --- | --- | --- | --- |
| B1 | 33 | 1.77 | 40.00 | 13.15 | 44.26 | 3.31 |
| T1 | 39 | 2.25 | 43.00 | 4.93 | 49.49 | 3.77 |
| B2 | 43 | 2.04 | 54.00 | 11.18 | 55.69 | 3.53 |
| T2 | 49 | 1.94 | 66.50 | 16.08 | 65.98 | 4.07 |
| B3 | 34 | 1.76 | 35.25 | 2.19 | 37.92 | 3.05 |
| T3 | 31 | 1.46 | 31.75 | 1.62 | 33.48 | 2.83 |
| B4 | 27 | 1.11 | 30.00 | 4.80 | 32.64 | 2.69 |
| T4 | 18 | 0.56 | 21.00 | 11.66 | 21.43 | 2.33 |
| B5 | 22 | 1.36 | 22.60 | 1.77 | 23.43 | 2.27 |
| T5 | 15 | 1.70 | 15.50 | 3.74 | 17.94 | 1.62 |
| B6 | 9 | 1.76 | 9.50 | 3.74 | 11.94 | 1.51 |
| T6 | 8 | 1.15 | 8.00 | NaN | 8.64 | 1.31 |
| Sample A | 40 | 1.05 | 47.85 | 8 | 48.82 | 3.26 |
| Sample B | 76 | 2.26 | 103 | 15.61 | 109.45 | 5.76 |
| Sample A16S | 32 | 2.03 | 77 | NaN | 41.91 | 3.16 |
| Sample B16S | 41 | 2.36 | 63.5 | 59.58 | 49.44 | 3.36 |
